# Epidemiology, Risk Factors, and Outcomes of Neutropenic Enterocolitis in Onco-Hematological Patients According to Chemotherapy Regimen

**DOI:** 10.1093/cid/ciaf134

**Published:** 2025-03-20

**Authors:** Anne-Sophie Brunel, Claire Seydoux, Sabine Schmidt, Siham Ahlyege, Aurélie Guillet, Katerina Mandralis, Mapi Fleury, Anne Cairoli, Sabine Blum, Olivier Spertini, Oscar Marchetti, Mathilde Gavillet, Pierre-Yves Bochud

**Affiliations:** Infectious Diseases Service, Department of Medicine, Lausanne University Hospital (CHUV) and University of Lausanne (UNIL), Lausanne, Switzerland; Infectious Diseases Service, University Hospital of Besançon, Besançon, France; Service and Central Laboratory of Hematology, Department of Oncology and Department of Laboratories and Pathology, Lausanne University Hospital (CHUV) and Lausanne University, Lausanne, Switzerland; Department of Diagnostic and Interventional Radiology, Lausanne University Hospital (CHUV) and University of Lausanne (UNIL), Lausanne, Switzerland; Service and Central Laboratory of Hematology, Department of Oncology and Department of Laboratories and Pathology, Lausanne University Hospital (CHUV) and Lausanne University, Lausanne, Switzerland; Infectious Diseases Service, Department of Medicine, Lausanne University Hospital (CHUV) and University of Lausanne (UNIL), Lausanne, Switzerland; Department of Diagnostic and Interventional Radiology, Lausanne University Hospital (CHUV) and University of Lausanne (UNIL), Lausanne, Switzerland; Department of Oncology, Lausanne University Hospital (CHUV) and Lausanne University, Lausanne, Switzerland; Service and Central Laboratory of Hematology, Department of Oncology and Department of Laboratories and Pathology, Lausanne University Hospital (CHUV) and Lausanne University, Lausanne, Switzerland; Service and Central Laboratory of Hematology, Department of Oncology and Department of Laboratories and Pathology, Lausanne University Hospital (CHUV) and Lausanne University, Lausanne, Switzerland; Service and Central Laboratory of Hematology, Department of Oncology and Department of Laboratories and Pathology, Lausanne University Hospital (CHUV) and Lausanne University, Lausanne, Switzerland; Infectious Diseases Service, Department of Medicine, Lausanne University Hospital (CHUV) and University of Lausanne (UNIL), Lausanne, Switzerland; Department of Medicine, Ensemble Hospitalier de la Côte, Morges, Switzerland; Service and Central Laboratory of Hematology, Department of Oncology and Department of Laboratories and Pathology, Lausanne University Hospital (CHUV) and Lausanne University, Lausanne, Switzerland; Infectious Diseases Service, Department of Medicine, Lausanne University Hospital (CHUV) and University of Lausanne (UNIL), Lausanne, Switzerland

**Keywords:** neutropenic enterocolitis, chemotherapy, hematological malignancies, autologous hematopoietic stem cell transplantation, acute leukemia

## Abstract

**Background:**

While neutropenic enterocolitis (NEC) is a well-known life-threatening complication during intensive chemotherapy, its incidence, impact, and outcome on specific at-risk populations remain ill defined.

**Methods:**

We report 178 NEC episodes during 1963 myeloablative chemotherapy courses among 1259 adult patients with acute myeloid (AML) or lymphoblastic (ALL) leukemia or receiving autologous hematopoietic stem cell transplantation (auto-HCT) for lymphoma or multiple myeloma. Risk factors were assessed by multivariate logistic regression models.

**Results:**

Most NEC cases (93.3%) occurred during AML induction (n = 92; 13.8% of chemotherapy course) and auto-HCT (n = 74; 9.5%). Independent risk factors for NEC during AML induction included high-dose corticosteroids (OR = 2.07; 95% CI: 1.29–3.30; *P* = .002), elevated circulating blasts at the time of diagnosis (>50 Giga/L; OR = 2.02; 95% CI: 1.15–3.56; *P* = .02), and use of azacitidine (OR = 2.45; 95% CI: 1.01–5.90; *P* = .05); purine-based regimens (eg, FLAG-Ida) were an independent protective factor (OR = .27; 95% CI: .15–.47; *P* < .001). Independent risk factors after auto-HCT included BEAM (carmustine, etoposide, cytarabine, and melphalan) versus another conditioning protocol (OR = 3.28; 95% CI: 1.98–5.43; *P* < .001) and age (OR = 1.03/year; 95% CI: 1.01–1.06; *P* = .007). For both AML induction and auto-HCT, NEC was associated with longer hospitalization (*P* = .03 and <.001), sepsis (quick SOFA ≥2; *P* = .03 and <.001), fungemia (*P* < .001 and *P* = .01), and intensive care admission (*P* = .03 and <.001, respectively). NEC was associated with increased in-hospital mortality during AML induction (6.5% vs 2.4%; *P* = .04) but not during auto-HCT (*P* = .3).

**Conclusions:**

The incidence of NEC depended on chemotherapeutic regimens, with higher occurrence during standard “7 + 3” AML induction and BEAM conditioning for auto-HCT. NEC was associated with longer hospitalization and increased morbidity, but 30-day mortality was lower than previously reported.

## Teaser

The incidence of neutropenic enterocolitis (NEC) highly depends on chemotherapy regimen used for acute myeloid leukemia induction and autologous hematopoietic stem cell transplantation. NEC is associated with longer hospitalization and increased morbidity, but overall mortality is lower than anticipated.

Neutropenic enterocolitis (NEC) is known as a life-threatening, necrotizing bowel inflammation occurring primarily in neutropenic patients undergoing intensive chemotherapy for the treatment of hematological malignancies [1, 2]. Pathophysiological events leading to NEC result from the combined action of chemotherapy-induced mucosal damage, coagulation disorders, and profound neutropenia, promoting bacterial and fungal translocation of the resident flora [2, 3].

The clinical manifestations of NEC overlap with those seen in other abdominal conditions, such as colitis due to *Clostridioides difficile,* cytomegalovirus (CMV) or ischemia, appendicitis, Ogilvie syndrome, or gastrointestinal graft-versus-host disease (GvHD) [2, 4]. The increasing use of computed tomography (CT) has improved our ability to distinguish NEC from the other digestive syndromes. Current diagnostic criteria proposed by Gorschlüter et al [5] include an absolute neutrophil count (ANC) of less than 500 × 10^6^ cells/L, fever greater than 38.3°, and bowel wall thickening (BWT) greater than 4 mm with a bowel length more than 30 mm detected by abdominal ultrasound or CT, in the absence of any other cause. The 4-mm cutoff is considered reliable as it is very rare in noninflammatory bowel disease after chemotherapy.

In the absence of a clear definition, the epidemiology of NEC has remained ill defined, with reported incidence ranging from 6% to 46% [2, 5–8]. Recent cytotoxic chemotherapy is the most reported risk factor [2, 9]. Pre-existing bowel abnormalities and prior episodes of NEC appear to increase the risk of relapse [2]. Altogether, patient heterogeneity and the absence of multivariate models in most studies make it difficult to determine which factors are independently associated with NEC.

The management of NEC relies mainly on expert opinion [5]. Medical treatment usually combines broad-spectrum antibiotics active against gram-negative bacteria and anaerobes [10], supportive therapy (bowel rest, intravenous fluids, parenteral nutrition), and granulocyte colony-stimulating factor (G-CSF); the benefit of empirical antifungal therapy in non–critically ill neutropenic patients with NEC is not clearly established [10]. While surgery was initially considered for all cases [11, 12], its current use is controversial [13, 14]. The mortality rate is high, ranging from 10% to 63% [3, 5, 7, 15–19].

The goals of this study were to determine the incidence, outcomes, and risk factors for NEC in a cohort of prospectively collected onco-hematological patients.

## METHODS

This study was conducted in a prospectively collected cohort of patients aged 18 years and older with hematological malignancies hospitalized within the isolation unit of Lausanne University Hospital between 1 January 2007 and 31 December 2023 (MINCO registry). It was approved by the local Ethics Committee (Swissethics 2017-01975).

### Definitions

Patients with acute myeloid leukemia (AML) or myelodysplastic syndrome with increased blasts 2 (MDS-IB2) were treated with cytarabine plus anthracycline induction according to the standard arm in ongoing K trials of the Dutch-Belgian Hemato-Oncology Cooperative Group (HOVON) and the Swiss Group for Clinical Cancer Research (SAKK) [20] or purine-based chemotherapy (FLAG-Ida (fludarabine, cytarabine, G-CSF, and idarubicine), CLAG-Ida (cladribine, cytarabine, G-CSF, and idarubicine)) [21–25] (Supplementary Table 1). Patients with acute lymphoblastic leukemia (ALL) were treated according to ongoing protocols of the Group for Research in Adult Acute Lymphoblastic Leukemia (GRAALL) [26]. Conditioning chemotherapy regimens for autologous hematopoietic stem cell transplantation (auto-HCT) were stratified as "BEAM (carmustine, etoposide, cytarabine, and melphalan) protocol" for patients with lymphoma [15] and as “other conditioning regimens,” including melphalan for multiple myeloma (MM), carmustine and thiotepa for central nervous system lymphoma, and busulfan and cyclophosphamide, for patients with AML [15]. Allogenic HCT is not performed in this hospital.

Febrile neutropenia (FN) was defined by the presence of an ANC less than 500 cells/mm^3^ and a tympanic temperature of 38.5°C or greater or of 38.0°C or greater measured twice over a 2-hour period. During each episode, the investigations systematically included sets of blood cultures, cultures from urine and any suspected site of infection, and a chest X-ray. Stool cultures to check for *Salmonella*, *Shigella*, or *Campylobacter* species and testing for *C difficile* toxins were routinely performed in cases of diarrhea, as well as CMV testing in patients with relapsed acute leukemia and a history of allogeneic HCT. Broad-spectrum empiric antibiotic therapy (cefepime +/− metronidazole or piperacillin-tazobactam) is promptly started after microbiological sampling at each episode of FN. Antifungal prophylaxis during neutropenia was prescribed according to international recommendations [10, 27].

### Data Collection

Data were extracted from the MINCO registry implemented in SecuTrial (InterActive Systems GmbH, version 5.5.0.25 (2025), Berlin), which contains an extensive range of prospectively collected data, including demographics, duration of neutropenia, characteristics of infections, treatments, outcomes, and sequential Sepsis-related Organ Failure Assessment (SOFA) and quick SOFA (qSOFA) scores [28]. All data were systematically collected by trained study nurses in a dedicated case-report form, reviewed by senior Infectious Diseases physicians and classified as microbiologically documented infection (MDI), clinically documented infection (CDI), or fever of unknown origin (FUO). Prognosis for AML was described according to the 2017 European Leukemia Network genetic categories [29]. Death occurring after hospitalization was obtained from the official death registry. The analyses were performed by chemotherapy cycles (each corresponding to a single chemotherapy and neutropenia episode), as the main risk conditions change at each cycle. The total dose of corticosteroids received during the chemotherapy episode is calculated in milligrams of prednisone equivalent [30].

The diagnosis of NEC was established when patients with FN had clinical signs and/or symptoms suggestive of abdominal infection (abdominal distension, tenderness and pain, diarrhea, vomiting, and/or bloody stools), with the presence of BWT greater than 4 mm (measured on axial CT images) over more than 30 mm of length (measured on coronal CT images) in any bowel segment, as proposed by Gorschlüter et al [5], in the absence of other well-recognized entities on CT (appendicitis, diverticulitis, cholecystitis, cholangitis, GvHD, ischemic colitis) or alternative diagnoses including enteric pathogens (such as *C difficile* and CMV-associated colitis). All abdominal CT examinations were reviewed by a radiologist with more than 20 years of practical experience in gastrointestinal imaging. The presence or absence of NEC was sought in the 4 quadrants of the small bowel (right upper, left upper, right lower, left lower) and the different segments of colon (cecum, ascending colon, transverse colon, descending colon, sigmoid, rectum). The maximal wall thickness for each segment and the percentage of involvement of the analyzed bowel segment were measured. Episodes of NEC occurring within 10 days of *C difficile* colitis were excluded.

### Statistical Analysis

Statistical analysis was performed using Stata version 18.0 software (StataCorp, College Station, TX). Risk factors for developing NEC were assessed in homogeneous groups of patients by using univariate and multivariate logistic regression models. Factors that were or tended to be associated with the endpoint on univariate analysis were entered into multivariate analysis and selected by using backward stepwise regression. Kaplan-Meier survival analyses were performed using the STS graph function with a landmark approach. Differences were considered significant for *P* values less than .05.

## RESULTS

### Study Population

A total of 1963 chemotherapy courses were analyzed in 1259 patients (Table 1). The patients’ median age was 58 years (Percentile 75 minus percentile 25 [P75-P25]: 17 years) and 60.0% were men. Most frequent malignancies were AML and MDS-IB2 (n = 379; 30.1%), MM (n = 374; 29.7%), lymphoma (n = 351; 27.9%), and ALL (n = 80; 6.4%). Of the 1963 chemotherapy courses, 665 were for the treatment of AML (“AML population,” including 231 first and 180 second standard inductions and 254 purine-based inductions or salvage) and 776 were conditioning regimen for auto-HCT (“auto-HCT population,” including 289 with a BEAM and 487 with another conditioning protocol; eg, melphalan). The other chemotherapy episodes included 47 ALL inductions and 475 chemotherapies for other hematological malignancies (detailed in Supplementary Table 2).

**Table 1.** Characteristics of the Study Population

Characteristics	Entire Population		Chemotherapy for AML		Autologous HCT	
	n	(%)	n	(%)	n	(%)
Number of chemotherapy courses	1963	…	665	…	776	…
Number of patients	1259	…	324	…	726	…
Patients demographics^a^						
Median (P75-P25) age, y	58	(17)	57	(18)	57	(14)
Gender, male	755	(60.0)	193	(59.6)	439	(60.5)
Ethnicity, Caucasian	1159	(92.1)	311	(96.0)	654	(90.1)
Chronic health conditions^a^						
Cardiac insufficiency	124	(9.8)	37	(11.4)	70	(9.6)
Pulmonary disease	103	(8.2)	32	(9.9)	61	(8.4)
Diabetes mellitus	106	(8.4)	18	(5.6)	62	(8.5)
Neurologic disease	72	(5.7)	14	(4.3)	36	(5.0)
Chronic renal insufficiency	65	(5.2)	10	(3.1)	42	(5.8)
Underlying malignancy^a^						
AML or MDS-IB2	379	(30.1)	291	(89.8)	25	(3.4)
MM	374	(29.7)	…	…	370	(51.0)
Lymphoma	351	(27.9)	…	…	317	(43.7)
ALL	80	(6.4)	12	(3.7)	4	(0.6)
Other	75	(6.0)	21	(6.5)	10	(1.4)
Chemotherapy regimens						
AML-induction						
Standard protocols^b^	411	(20.9)	411	(61.8)	…	…
Purine-based chemotherapy^c^	254	(12.9)	254	(38.2)	…	…
Auto-HCT						
BEAM conditioning^d^	289	(14.7)	…	…	289	(37.2)
Non-BEAM conditioning^e^	487	(24.8)	…	…	487	(62.8)
ALL—induction	47	(2.4)	…	…	…	…
Other^f^	475	(24.2)	93	(14.0)	…	…
Previous allogeneic HCT	69	(3.5)	44	(6.6)	1	(0.1)
Days in hospital, median (P75-P25)	26	(16)	33	(16)	20	(7)
Days of neutropenia, median (P75-P25)	10	(13)	21	(14)	7	(3)
Neutropenic enterocolitis	178	(9.1)	92	(13.8)	74	(9.5)

Continuous variables are described using medians and IQRs and categorical variables using numbers and percentages (%).

Abbreviations: ALL, acute lymphoblastic leukemia; AML, acute myeloid leukemia; Auto-HCT, autologous hematopoietic stem cell transplantation; BEAM, carmustine, etoposide, cytarabine, and melphalan; G-CSF, granulocyte colony-stimulating factor; HCT, hematopoietic stem cell transplantation; MDS-IB2, myelodysplastic syndrome with increased blasts 2; MM, multiple myeloma; P75-P25 stands for percentile 75 minus percentile 25.

^a^Demographic characteristics, chronic health conditions, and underlying malignancies are reported by patient (not by chemotherapy course).

^b^All standard induction regimens included standard-dose cytarabine with anthracyclines (mostly idarubicine or daunorubicine, “7 + 3”) for the first induction cycle and high-dose cytarabine +/− amsacrine or daunorubicine for the second induction cycle, according to specific protocols of the Dutch-Belgian Hemato-Oncology Cooperative Group (HOVON) and the Swiss Group for Clinical Cancer Research (SAKK) [20].

^c^Purine-based chemotherapy regimens included FLAG(-Ida) (fludarabine, high-dose cytarabine, G-CSF, +/− idarubicine) and CLAG(-Ida) (cladribine, high-dose cytarabine, G-CSF, +/− idarubicine) protocols [21–25].

^d^BEAM regimen included carmustine, etoposide, cytarabine, and melphalan [15].

^e^Other conditioning regimens included melphalan or other chemotherapies [15].

^f^Other chemotherapies included those administered for multiple myeloma or non-Hodgkin's lymphoma/Hodgkin's lymphoma, or consolidation regimens for acute leukemia.

### Characteristics of Neutropenic Enterocolitis

**Figure 1.**
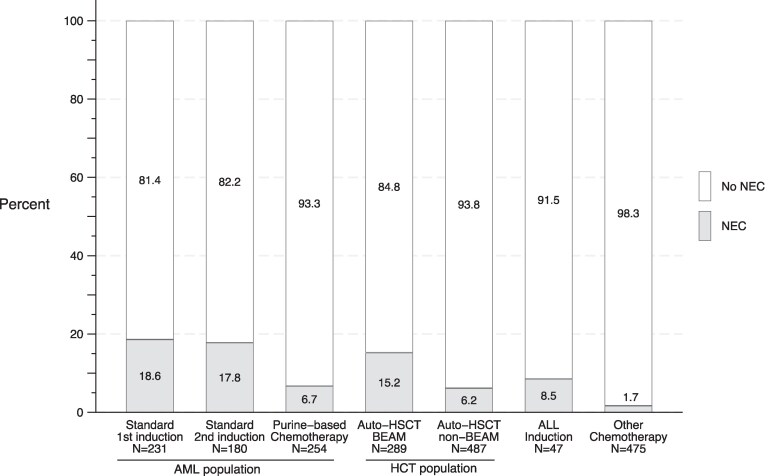
Incidence of NEC in onco-hematological patients according to different chemotherapy regimens. NEC was defined by the presence of clinical signs and/or symptoms suggestive of abdominal infection, in the absence of an alternative diagnosis, and bowel wall thickening >4 mm (measured on axial CT images) in any bowel segment. Abbreviations: ALL, acute lymphoblastic leukemia; AML, acute myeloid leukemia; auto-HSCT, autologous hematopoietic stem cell transplantation; BEAM, carmustine, etoposide, cytarabine, and melphalan; CT, computed tomography; NEC, neutropenic enterocolitis.

A total of 178 episodes of NEC were diagnosed during 1963 courses of chemotherapy (9.1%) (Supplementary Table 3). The vast majority (93.3% of NEC episodes) occurred in the AML (n = 92; 13.8% of courses) and the auto-HCT (n = 74; 9.5%) populations. Yet, the incidence of NEC varied within these 2 groups after stratification according to specific chemotherapeutic regimen (Figure 1). In the AML population, this incidence was much higher during induction with a standard (“7 + 3”) protocol (18.6% for the first and 17.8% for the second induction) than during purine-based chemotherapy (6.7%). In the auto-HCT population, it was higher among patients receiving a BEAM protocol (15.2%) compared with those receiving another protocol (6.2%). The incidence of NEC was 8.5% during ALL induction and only 1.7% during other chemotherapies.

**Figure 2.**
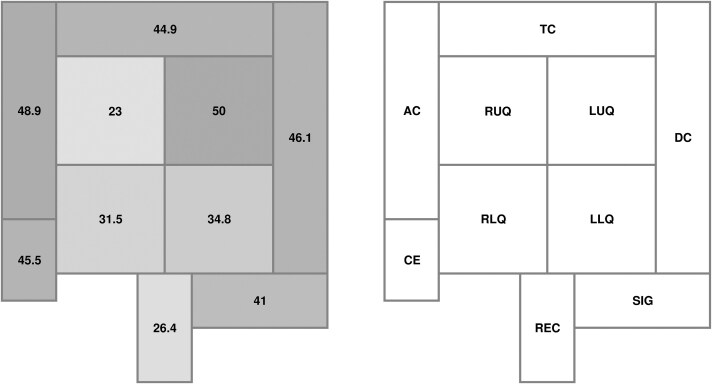
Anatomic localization of NEC in onco-hematological patients. Values within the different segments represent the percentage of NEC patients for whom a wall thickening >4 mm was measured on axial CT images. Abbreviations: AC, ascendent colon; CE, cecum; CT, computed tomography; DC, descendent colon; LLQ, left lower abdominal quadrant; LUQ, left upper abdominal quadrant; NEC, neutropenic enterocolitis; REC, rectum; RLQ, right lower abdominal quadrant; RUQ, right upper abdominal quadrant; SIG, sigmoid; TC, transverse colon.

The analysis of abdominal CT examinations revealed that NEC involved more than 1 intestinal segment in 91% of cases (median number of segments = 3, IQR = 3), with the small intestine situated in the upper left abdominal quadrant (50.0% of cases) and the ascending colon (48.9%) being the most frequently affected localization (Figure 2). The median maximal BWT ranged from 8 mm (IQR = 3 mm) in the right lower quadrant of the small intestine to 15 mm (IQR = 8 mm) in the cecum.

### Risk Factors

In the AML population, independent risk factors for NEC included high-dose corticosteroids (>100 mg prednisone equivalent; odds ratio [OR] = 2.07; 95% CI: 1.29–3.30; *P* = .002), elevated circulating blast count at the time of AML diagnosis (>50 G/L; OR = 2.02; 95% CI: 1.15–3.56; *P* = .02) (Table 2), and concomitant use of azacitidine, a pyrimidine analogue (OR = 2.45; 95% CI: 1.01–5.90; *P* = .05). Purine-based chemotherapy instead of standard induction was an independent protective factor (OR = .27; 95% CI: .15–.47; *P* < .001). Additional risk factors emerged when the analyses were further limited to patients receiving standard AML induction, the group with the highest incidence of NEC, by entering specific regimens into the multivariate models (Table 3). In addition to high-dose corticosteroids (prednisone equivalent >100 mg; OR = 2.77; 95% CI: 1.57–4.88; *P* < .001), elevated circulating blast counts (OR = 2.64; 95% CI: 1.37–5.08; *P* = .004 for blasts 20–50 G/L; OR = 2.25; 95% CI: 1.08–4.69; *P* = .03 for blasts >50 G/L, compared with blasts <20 G/L), and the use of azacitidine (OR = 3.29; 95% CI: 1.03–10.5; *P* = .04), the analysis also identified a previous NEC (OR = 3.65; 95% CI: 1.48–9.04; *P* = .005), as well as the use of regimens containing idarubicine (OR = 2.66; 95% CI: 1.31–5.41; *P* = .007) and amsacrine (OR = 3.24; 95% CI: 1.54–6.84; *P* = .002), versus cytarabine alone or in combination with daunorubicine, as independent risk factors for NEC.

**Table 2.** Risk Factors for Neutropenic Enterocolitis in Acute Myeloid Leukemia Induction

Characteristics	All AML Induction Courses (N = 665)									
	No NEC (n = 573)		NEC (n = 92)		Univariate			Multivariate^a^		
	n	(%)	n	(%)	OR	(95% CI)	*P*	OR	(95% CI)	*P*
Age, median (P75-P25), y	57	(19.3)	54	(17.2)	.99	(.98–1.01)	.5	…	…	
Gender, male	348	(60.7)	57	(62.0)	1.05	(.67–1.66)	.8	…	…	
Ethnicity, Caucasian	548	(95.6)	89	(96.7)	1.35	(.40–4.58)	.6	…	…	
Chronic health conditions										
Cardiac insufficiency	68	(11.9)	11	(12.0)	1.01	(.51–1.99)	.9	…	…	
Pulmonary disease	67	(11.7)	6	(6.5)	.53	(.22–1.25)	.1	…	…	
Chronic renal failure	24	(4.2)	1	(1.1)	.25	(.03–1.88)	.2	…	…	
Neurological disease	21	(3.7)	2	(2.2)	.58	(.13–2.53)	.5	…	…	
Diabetes mellitus	35	(6.1)	4	(4.4)	.70	(.24–2.01)	.5	…	…	
Tobacco use	172	(30.0)	30	(32.6)	1.13	(.70–1.81)	.6	…	…	
Prognostic score of AML^b^										
Favorable risk	92	(20.2)	16	(20.3)	Ref	…		…	…	
Intermediate risk	179	(39.3)	27	(34.2)	.87	(.44–1.69)	.7	…	…	
Poor risk	185	(40.6)	36	(45.6)	1.12	(.59–2.12)	.7	…	…	
Blasts counts at admission										
<20 Giga/L	380	(66.3)	49	(53.3)	Ref	…		…	…	
20–50 Giga/L	120	(20.9)	22	(23.9)	1.42	(.83–2.45)	.2	…	…	
>50 Giga/L	73	(12.7)	21	(22.8)	2.23	(1.26–3.94)	.006	2.02	(1.15–3.56)	.02
Chemotherapy regimen^c^										
Standard induction	336	(58.6)	75	(81.5)	Ref	…		…	…	
Purine-based chemotherapy	237	(41.4)	17	(18.5)	.32	(.19–.56)	<.001	.27	(.15–.47)	<.001
Duration of agranulocytosis										
<10 d	35	(6.1)	4	(4.4)	Ref	…		…	…	
11–25 d	336	(58.6)	57	(62.0)	1.48	(.51–4.34)	.5	…	…	
>25 d	202	(35.3)	31	(33.7)	1.34	(.45–4.04)	.6	…	…	
Other agents or conditions										
Azacitidine	22	(3.8)	8	(8.7)	2.39	(1.03–5.53)	.04	2.45	(1.01–5.90)	.05
Hydroxycarbamide	81	(14.1)	21	(22.8)	1.80	(1.05–3.08)	.03	…	…	
Corticosteroids >100 mg^d^	260	(45.4)	56	(60.9)	1.87	(1.19–2.94)	.006	2.07	(1.29–3.30)	.002
Anti-*Candida* prophylaxis	439	(76.6)	71	(77.2)	1.03	(.61–1.74)	.9	…	…	
G-CSF	459	(80.1)	74	(80.4)	1.02	(.59–1.78)	.9	…	…	
Previous NEC	55	(9.6)	11	(12.0)	1.28	(.64–2.55)	.5	…	…	

Continuous variables are described using medians and IQRs, and categorical variables are described using numbers and proportions (%). Characteristics are reported by chemotherapy episode.

Abbreviations: AML, acute myeloid leukemia; CI, confidence interval; G-CSF, granulocyte-colony stimulating factor; NEC, neutropenic enterocolitis; OR, odds ratio; P75-P25 stands for percentile 75 minus percentile 25; Ref, reference.

^a^Variables with a *P* value <.1 were entered into multivariable models and subsequently selected by using the stepwise program implemented in Stata (StataCorp, College Station, TX), with backward removal of variables with a *P* value <.1. Hydroxycarbamide was excluded from the analysis due to collinearity with high circulating blast count.

^b^Prognostic score classification according to the 2017 European Leukemia Network [29]. Data were missing for 130 patients.

^c^All standard induction regimens included standard-dose cytarabine with anthracyclines (mostly idarubicine or daunorubicine, “7 + 3”) for the first induction cycle and high-dose cytarabine +/− amsacrine or daunorubicine for the second induction cycle, according to specific protocol of the Dutch-Belgian Hemato-Oncology Cooperative Group (HOVON) and the Swiss Group for Clinical Cancer Research (SAKK) [20]. Purine-based chemotherapy regimens included FLAG(-Ida) (fludarabine, high-dose cytarabine, G-CSF, +/− idarubicine) and CLAG(-Ida) (cladribine, high-dose cytarabine, G-CSF, +/− idarubicine) protocols [21–25].

^d^Total doses of corticosteroid during chemotherapy episode were calculated in prednisone equivalents: hydrocortisone (× 0.3), prednisolone (× 1), methylprednisolone (× 1.25), dexamethasone or betamethasone (× 6.7) [30].

**Table 3.** Risk Factors for Neutropenic Enterocolitis in Acute Myeloid Leukemia Induction (Subgroup of Patients Treated With “Standard” Induction Protocols)

Characteristics	All Induction Courses (N = 411)									
	No NEC (n = 336)		NEC (n = 75)		Univariate (n = 411)			Multivariate^d^ (n = 411)		
	n	(%)	n	(%)	OR	(95% CI)	*P*	OR	(95% CI)	*P*
Age, median (P75-P25), y	56	(19)	55	(18)	1.00	(.98–1.02)	.8	…	…	
Gender, male	196	(58.3)	47	(62.7)	1.20	(.72–2.01)	.5	…	…	
Ethnicity, Caucasian	321	(95.5)	73	(97.3)	1.71	(.38–7.62)	.5	…	…	
Chronic health conditions										
Cardiac insufficiency	34	(10.1)	10	(13.3)	1.37	(.64–2.91)	.4	…	…	
Pulmonary disease	36	(10.7)	5	(6.7)	.60	(.23–1.57)	.3	…	…	
Chronic renal failure	13	(3.9)	1	(1.3)	.34	(.04–2.61)	.3	…	…	
Neurological disease	12	(3.6)	1	(1.3)	.36	(.05–2.85)	.3	…	…	
Diabetes mellitus	18	(5.4)	3	(4.0)	.74	(.21–2.57)	.6	…	…	
Tobacco use	101	(30.1)	26	(34.7)	1.23	(.73–2.10)	.4	…	…	
Prognostic score of AML^a^										
Favorable risk	59	(21.3)	14	(20.6)	Ref	…		…	…	
Intermediate risk	116	(41.9)	25	(36.8)	.91	(.44–1.88)	.8	…	…	
Poor risk	102	(36.8)	29	(42.7)	1.20	(.59–2.45)	.6	…	…	
Blasts at admission										
<20 Giga/L	245	(72.9)	39	(52.0)	Ref	…		…	…	
20–50 Giga/L	51	(15.2)	21	(28.0)	2.59	(1.41–4.76)	.002	2.64	(1.37–5.08)	.004
>50 Giga/L	40	(11.9)	15	(20.0)	2.36	(1.19–4.66)	.014	2.25	(1.08–4.69)	.03
HOVON-based regimen^b^										
Cytarabine alone	35	(10.4)	6	(8.0)	Ref	…		…	…	
Cytarabine with daunorubicine	98	(29.2)	15	(20.0)	.89	(.32–2.48)	.8	…	…	
Cytarabine with idarubicine	132	(39.3)	32	(42.7)	1.41	(.55–3.65)	.5	2.66	(1.31–5.41)	.007
Cytarabine with amsacrine	71	(21.1)	22	(29.3)	1.81	(.67–4.86)	.2	3.24	(1.54–6.84)	.002
Second (vs first) induction	148	(44.1)	32	(42.7)	.95	(.57–1.57)	.8	…	…	
Duration of agranulocytosis										
<10 d	15	(4.5)	2	(2.7)	Ref	…		…	…	
11–25 d	177	(52.7)	48	(64.0)	2.03	(.45–9.20)	.4	…	…	
>25 d	144	(42.9)	25	(33.3)	1.30	(.28–6.05)	.7	…	…	
Other agents or conditions										
Azacitidine	10	(3.0)	6	(8.0)	2.83	(1.00–8.06)	.05	3.29	(1.03–10.5)	.04
Hydroxycarbamide	50	(14.9)	19	(25.3)	1.94	(1.06–3.54)	.03	…	…	
Corticosteroids >100 mg^c^	129	(38.4)	44	(58.7)	2.28	(1.37–3.79)	.002	2.77	(1.57–4.88)	<.001
Anti-*Candida* prophylaxis	264	(78.6)	57	(76.0)	.86	(.48–15.6)	.6	…	…	
G-CSF	232	(69.1)	57	(76.0)	1.42	(.80–2.53)	.2	…	…	
Previous NEC	20	(6.0)	10	(13.3)	2.43	(1.09–5.44)	.03	3.65	(1.48–9.04)	.005

Continuous variables are described using medians and IQRs, and categorical variables are described using numbers and proportions (%). Characteristics are reported by chemotherapy episode.

Abbreviations: AML, acute myeloid leukemia; CI, confidence interval; G-CSF, granulocyte-colony stimulating factor; HOVON, Dutch-Belgian Hemato-Oncology Cooperative Group; NEC, neutropenic enterocolitis; OR, odd ratio; P75-P25 stands for percentile 75 minus percentile 25; Ref, reference.

^a^Prognostic score classification according to the 2017 European Leukemia Network [29]. Data were missing in 66 patients.

^b^All standard inductions regimens included standard-dose cytarabine with anthracyclines (mostly idarubicine or daunorubicine, “7 + 3”) for the first induction cycle and high-dose cytarabine +/− amsacrine or daunorubicine for the second induction cycle, according to the number of the HOVON/SAKK protocol [20].

^c^Total doses of corticosteroid during chemotherapy episode were calculated in prednisone equivalents: hydrocortisone (× 0.3), prednisolone (× 1), methylprednisolone (× 1.25), dexamethasone or betamethasone (× 6.7) [30].

^d^Variables with a *P* value <.25 were entered into multivariable models and subsequently selected by using the stepwise program implemented in Stata (StataCorp, College Station, TX), with backward removal of variable with a *P* value <.1. Hydroxycarbamide was excluded from the analysis due to collinearity with high circulating blast count.

In the auto-HCT population, the only 2 factors independently associated with NEC were conditioning with BEAM protocol (OR = 3.28; 95% CI: 1.98–5.43; *P* < .001) (Table 4) versus another regimen and increasing age (OR = 1.03 per year; 95% CI: 1.01–1.06; *P* = .007). In the ALL population, no significant risk factor for NEC was identified, but the number of such episodes was quite small (n = 4) (Supplementary Table 4). In the other chemotherapies, the only independent risk factor for NEC was previous NEC (OR = 5.76; 95% CI: 1.08–30.73; *P* < .001) (Supplementary Table 5) despite the small number of NEC cases (n = 8).

**Table 4.** Risk Factors for Neutropenic Enterocolitis in Autologous Hematopoietic Stem Cell Transplantation

Characteristics	All Auto-HCT Courses (N = 776)									
	No NEC (n = 702)		NEC (n = 74)		Univariate (n = 776)			Multivariate^a^ (n = 775)		
	n	(%)	n	(%)	OR	(95% CI)	*P*	OR	(95% CI)	*P*
Age, median (P75-P25), y	57	(13.7)	60	(10.4)	1.02	(1.00–1.04)	.1	1.03	(1.01–1.06)	.007
Gender, male	425	(60.5)	48	(64.9)	1.20	(.73–1.99)	.5	…	…	
Ethnicity, Caucasian	634	(90.3)	66	(89.2)	.88	(.41–1.92)	.8	…	…	
Chronic health conditions										
Cardiac insufficiency	65	(9.3)	10	(13.5)	1.53	(.75–3.13)	.2	…	…	
Pulmonary disease	58	(8.3)	7	(9.5)	1.16	(.51–2.64)	.7	…	…	
Chronic renal failure	39	(5.6)	6	(8.1)	1.50	(.61–3.67)	.4	…	…	
Neurological disease	36	(5.1)	3	(4.1)	.78	(.23–2.60)	.7	…	…	
Diabetes mellitus	63	(9.0)	4	(5.4)	.58	(.20–1.64)	.3	…	…	
Tobacco use	149	(21.2)	16	(21.6)	1.02	(.57–1.83)	.9	…	…	
Underlying disease										
Multiple myeloma	394	(56.1)	26	(35.1)	Ref	…		…	…	
Lymphoma^b^	273	(38.9)	44	(59.5)	2.44	(1.47–4.06)	.001	…	…	
Hodgkin lymphoma	225	(32.1)	35	(47.3)	2.36	(1.38–4.02)	.002	…	…	
Non-Hodgkin lymphoma	48	(6.8)	9	(12.2)	2.84	(1.26–6.42)	.012	…	…	
Other^c^	35	(5.0)	4	(5.4)	1.73	(.57–5.24)	.3	…	…	
Chemotherapy regimen^d^										
Non-BEAM	457	(65.1)	30	(40.5)	Ref	…		…	…	
BEAM	245	(34.9)	44	(59.5)	2.74	(1.68–4.46)	<.001	3.28	(1.98–5.43)	<.001
Duration of agranulocytosis										
0–10 d	666	(94.9)	66	(89.2)	Ref	…		…	…	
>10 d	36	(5.1)	8	(10.8)	2.24	(1.00–5.02)	.05	…	…	
Other agents or conditions										
Corticosteroids >250 mg^e^	483	(68.8)	57	(77.0)	1.52	(.86–2.67)	.1	…	…	
G-CSF	694	(98.9)	73	(98.7)	.84	(.10–6.82)	.9	…	…	
Previous NEC	2	(0.3)	0	(0)	…	…		…	…	

Continuous variables are described using medians and IQRs, and categorical variables are described using numbers and proportions (%). Characteristics are reported by 776 chemotherapy episodes in 726 patients.

Abbreviations: Auto-HCT, autologous hematopoietic stem cell transplantation; BEAM, carmustine, etoposide, cytarabine, and melphalan; CI, confidence interval; G-CSF, granulocyte-colony stimulating factor; NEC, neutropenic enterocolitis; OR, odd ratio; P75-P25 stands for percentile 75 minus percentile 25; Ref, reference.

^a^Variables with a *P* value <.2 were entered into multivariable models and subsequently selected by using the stepwise program implemented in Stata (StataCorp, College Station, TX), with backward removal of variable with a *P* value <.1.

^b^Non-Hodgkin lymphoma (n = 260) had an NEC incidence of 16% and Hodgkin lymphoma (n = 57) had an NEC incidence of 13%.

^c^Acute myeloid leukemia (n = 26), acute lymphoblastic leukemia (n = 6), chronic lymphoblastic leukemia (n = 1), POEMS (Polyneuropathy, Organomegaly, Endocrinopathy, M-protein, and Skin changes) syndrome (n = 2), myelodysplastic syndrome (n = 1), multifocal plasmocytoma (n = 1), amyloidosis (n = 1), lymphomatous granulomatosis (n = 1).

^d^BEAM regimen included carmustine, etoposide, cytarabine, and melphalan and was administered almost exclusively to patients with lymphoma (n = 285) [15]. Non-BEAM regimens included mostly melphalan (for multiple myeloma) (n = 428) and other chemotherapies (n = 59), including busulfan-melphalan (Bu-Mel; n = 26), carmustine-thiothepa (n = 26), cyclophosphamide-etoposide (n = 4), and cyclophosphamide total body irradiation (n = 3).

^e^Total doses of corticosteroid during chemotherapy episode were calculated in prednisone equivalents: hydrocortisone (× 0.3), prednisolone (× 1), methylprednisolone (× 1.25), dexamethasone or betamethasone (× 6.7) [30].

### Treatment and Outcome

Broad-spectrum antibiotics were administered to all NEC cases (100%), as an initial empirical therapy in 99 cases in whom NEC appeared as the first episode of neutropenic fever (55.6%) or as a switch from (n = 84; 47.2%), or continuation of (n = 15; 8.4%), ongoing antibiotics, when NEC was preceded by 1 or several episode(s) of neutropenic fever (Supplementary Table 3). Antifungals were administered in 119 out of 178 cases of NEC (66.9%), as an initial antifungal therapy (n = 49; 27.6%) or as a switch from (n = 18; 10.1%), or maintenance of (n = 52; 29.2%), antifungals previously given as prophylaxis or treatment. Abdominal surgery with resection was performed in 3 episodes of NEC, only 1 of whom had perforation.

**Figure 3.**
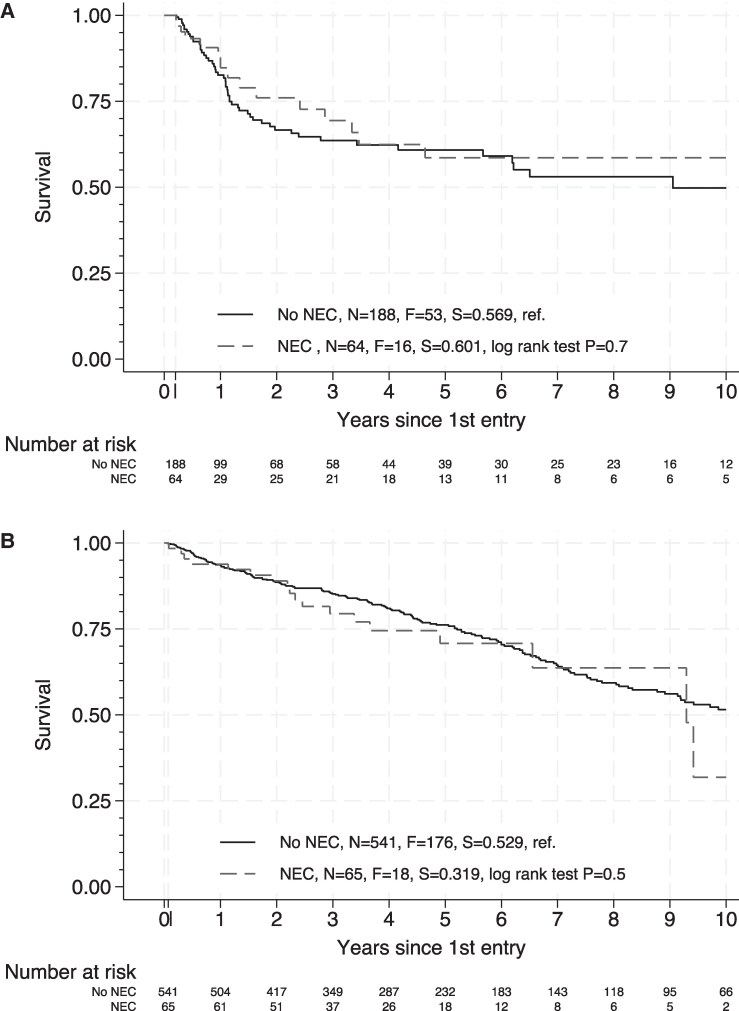
Overall survival analysis in hematological patients according to the presence or absence of NEC. We used a landmark analysis to estimate overall survival according to NEC, starting 10 weeks after the first AML induction (*A*) and 3 weeks after auto-HCT conditioning (*B*), with censoring when patients were hospitalized for a novel treatment, making them again at risk to develop NEC, including induction for new (after auto-HCT) or relapsed AML, auto-HCT (following AML induction or after a previous auto-HCT), or allo-HCT. The 3- and 10-week cutoffs (indicated by an arrow) were chosen since all NEC cases had occurred within 3 weeks after auto-HCT conditioning and within 10 weeks after the first AML induction (a duration encompassing neutropenic episodes of both the first and second induction), respectively. Long-term survival was assessable in 606 patients with auto-HCT and 252 patients with AML induction. N stands for number at risk, F for failure (death), and S for survival. Abbreviations: allo-HCT, allogenic hematopoietic stem cell transplantation; AML, acute myeloid leukemia; auto-HCT, autologous hematopoietic stem cell transplantation; NEC, neutropenic enterocolitis; ref, reference.

Neutropenic episodes complicated by NEC compared with those without NEC were characterized by a longer hospitalization (median [P75-25]: 34 [25] days vs 32 [17] days; *P* = .03 for AML induction; median [P75-P25]: 26 [8] days vs 20 [6] days; *P* < .001 for auto-HCT) (Table 5), a higher occurrence of sepsis (qSOFA ≥2: 19.6% vs 11.3%, *P* = .03, for AML induction; 23.0% vs 5.4%, *P* <.001, for auto-HCT; SOFA >10: 4.3% vs 1.0% for AML induction, *P* = .03; 4.1% vs 0.4%, *P* = .003 for auto-HCT), and a higher need for transfer to the intensive care unit (ICU) (14.1% vs 7.3%, *P* = .03, for AML induction; 18.9% vs 2.1%, *P* < .001, for auto-HCT). Neutropenic enterocolitis was associated with a higher rate of fungemia (6.5% vs 0.7%, *P* < .001, during AML induction; 4.1% vs 0.6%, *P* = .01, during auto-HCT), and during AML induction but not auto-HCT, a higher rate of bacteremia due to gram-positive cocci (38.0% vs 25.7%; *P* = .01). Finally, NEC was associated with a higher rate of in-hospital mortality during AML induction (6.5% vs 2.4%; *P* = .04), particularly after purine-based chemotherapy (17.6% vs 2.5%; *P* = .005) (Supplementary Table 6), but not during auto-HCT (*P* = .3). However, overall, long-term survival was not affected by NEC (log-rank test: *P* = .7 for AML and *P* = .5 for auto-HCT) (Figure 3).

**Table 5.** Comparative Outcomes of Patients With and Without Neutropenic Enterocolitis According to Chemotherapy Regimens

Characteristics	AML Induction					HCT				
	No NEC		NEC			No NEC		NEC		
	n	(%)	n	(%)	*P*	n	(%)	n	(%)	*P*
Chemotherapy episodes	573	…	92	…		702	…	74	…	
Days in hospital, median (P75-P25)	32	(17)	34	(25)	.03	20	(6)	26	(8)	<.001
Associated infections^a^										
Bacteremia	295	(51.5)	50	(54.3)	.6	157	(22.4)	17	(23.0)	.9
Gram-negative bacilli	128	(22.3)	20	(21.7)	.9	85	(12.1)	8	(10.8)	.7
Gram-positive cocci	147	(25.7)	35	(38.0)	.01	56	(8)	7	(9.5)	.7
Anaerobes	21	(3.7)	1	(1.1)	.2	4	(0.6)	1	(1.4)	.4
Polymicrobial	141	(24.6)	20	(21.7)	.6	88	(12.5)	9	(12.2)	.9
Fungemia	4	(0.7)	6	(6.5)	<.001	4	(0.6)	3	(4.1)	.01
Hepatosplenic candidiasis^b^	9	(1.6)	2	(2.2)	.7	…	…	…	…	
Invasive mold infection^c^										
Pulmonary	40	(7.0)	11	(12.0)	.1	1	(0.1)	…	…	
Digestive	1	(0.2)	1	(1.1)^d^	.2	…	…	…	…	
Sepsis severity scores^e^										
Quick SOFA ≥2^f^	65	(11.3)	18	(19.6)	.03	38	(5.4)	17	(23.0)	<.001
SOFA^g^										
<5	511	(89.2)	84	(91.3)	Ref	674	(96)	60	(81.1)	Ref
5–10	56	(9.8)	4	(4.3)	.1	25	(3.6)	11	(14.9)	<.001
>10	6	(1.0)	4	(4.3)	.03	3	(0.4)	3	(4.1)	.003
Transfer to ICU^h^	42	(7.3)	13	(14.1)	.03	14	(2.1)	14	(18.9)	<.001
Abdominal surgery	5	(0.9)	3	(2.2)	.07	…	…	1	(1.4)	
In-hospital all-cause mortality^i^	14	(2.4)	6	(6.5)	.04	3	(0.4)	1	(1.4)	.3

Continuous variables are described using medians and IQRs, and categorical variables are described using numbers and proportions (%). Characteristics are reported by NEC episodes.

Abbreviations: AML, acute myeloid leukemia; HCT, hematopoietic stem cell transplantation; ICU, intensive care unit; NEC, neutropenic enterocolitis; P75-P25 stands for percentile 75 minus percentile 25; Ref, reference; SOFA, Sequential Organ Failure Assessment.

^a^Infections identified during the entire hospital stay.

^b^In the standard induction AML group, 1/8 and 1/2 had concomitant fungemia in the no NEC group and NEC group, respectively. In the purine-based chemotherapy group, no concomitant fungemia was documented.

^c^Among invasive filamentous fungal infections, the number of invasive aspergillosis was 27/34 in the standard AML induction group and 16/25 in the purine-based chemotherapy group for AML.

^d^NEC was complicated by suspected or confirmed abdominal mold infections in 1 patient who died.

^e^Worse severity score during neutropenic episode.

^f^The Quick SOFA score [28] ranged from 0 to 3 according to the presence or absence of these criteria: systolic blood pressure ≤100 mmHg, tachypnea ≥22 breaths/min and Glasgow Coma Scale score ≤14 (1 point each); patients who did not develop fever and/or symptoms suggestive of sepsis were allocated the minimal score.

^g^Patients who did not develop fever and/or symptoms suggestive of sepsis are in the lower score group (<5).

^h^All causes of ICU admission during the hospital course.

^i^All causes of mortality; see Figure 2 for Kaplan-Meier survival estimates.

## DISCUSSION

Although NEC is considered to be a life-threatening condition, its exact incidence and outcome have long remained undefined, probably due to the lack of a standard definition, inhomogeneity, and limited size of study populations and retrospective nature of most reports [2, 5]. To our knowledge, this is the largest NEC cohort study and the first to stratify risk factor analyses by chemotherapeutic regimen.

Historically, NEC was associated with the use of cytarabine [6, 7, 9], which induces mucosal damages in the gastrointestinal tract [31]. Yet, this observation is not useful for predicting which patients with AML are likely to develop NEC, since this drug is part of virtually all regimens used for AML induction [6, 7, 9]. We show for the first time that the incidence of NEC can greatly vary among patients receiving cytarabine. The incidence of NEC was quite elevated after both a first (18.6%) and a second (17.8%) induction with a standard (“7 + 3”) regimen, while cytarabine doses greatly differ among these protocols (200 mg/m^2^/d, days 1–7, vs 2000 mg/m^2^/d, days 1–6, respectively). Furthermore, purine-based therapy (regardless of its use as front-line or salvage therapy) was associated with a lower rate of NEC compared with standard “7 + 3” induction despite elevated doses of cytarabine (2000 mg/m^2^/d, days 1–5 for FLAG). This suggests that cytarabine bowel toxicity may not much rely on its dosage but rather on factors such as the number of days it is administered, its daily infusion mode (1 vs 2 times per day, long vs short administration), and/or the type of associated chemotherapeutic drug. In standard AML induction, the incidence of NEC was higher when cytarabine was combined with idarubicine, a semi-synthetic glycoside analogue of daunorubicine with slightly different pharmacokinetic properties, or amsacrine compared with cytarabine alone or combined with daunorubicine, suggesting a potentiating role for these drugs, as previously reported with amsacrine [32]. The use of upfront G-CSF, which is systematic in purine-based regimens (eg FLAG-Ida), may have contributed to reduce neutropenia duration and the risk of NEC in this group. Nevertheless, G-CSF was not a significant protective factor for NEC during standard AML induction. The other relevant risk factor for NEC in our AML population was a previous NEC and the high level of circulating blasts (>50 Giga/L) at admission. A likely mechanism is the presence is leukemic infiltration of the bowel at the beginning of chemotherapy, which plays a role in the pathogenesis of NEC, as previously suggested [3], although this has not been confirmed histologically [33].

The second regimen associated with NEC was auto-HCT conditioning with a BEAM protocol, with a global incidence of 15% (16% for Non-Hodgkin lymphoma (NHL) and 13% for Hodgkin lymphoma (HL)); this is consistent with a smaller report of 25 episodes of NEC occurring in 179 BEAM recipients (14%), although the proportion of NEC differed according to the underlying disease in this study (19% for NHL and 9% for HL) [15]. In a recent prospective study of 129 patients with lymphoma conditioned with BEAM, NEC incidence was even higher (31%), but the diagnosis was obtained by ultrasonography, a technique that is more subjective and far less reproducible than CT [34]. Since the doses of melphalan and cytarabine are lower in the BEAM protocol than in the conditioning regimen for MM and purine-based AML regimens, respectively, NEC may result from a potentiating effect of the other drugs used in these regimens—namely, carmustine and etoposide.

This study is the first to identify steroids as a risk factor for NEC. Cumulative doses greater than 100 mg of prednisone equivalent significantly increased this risk during AML induction, and higher doses (>250 mg) also tended to do so during auto-HCT. A smaller study of 52 episodes of typhlitis failed to detect such an association, but the results are difficult to interpret in the absence of dose reporting [9]. Since steroids are increasingly administered within or in addition to chemotherapy regimens to prevent gastrointestinal side effects or to treat severe cutaneous and/or digestive damage secondary to high doses of cytarabine, the identification of their role as a risk factor for NEC is clinically relevant.

While earlier studies reported NEC mortality rates of more than 50% [5, 7], 30-day mortality rates ranged from 9% to 30% in more recent reports [3, 15–18, 35]. In-hospital mortality (6.5% for AML induction and 2.4% for auto-HCT) was even lower in our study. Such differences may be due to several factors. First, the literature is often limited to case reports or small case series, with a potential publication bias towards severe cases [5, 35, 36]. Second, clinical screening, diagnostic procedures, and criteria definitions for NEC have evolved and differ among centers, making it difficult to compare NEC incidence and mortality rates. Finally, close monitoring since the first sepsis signs, as illustrated by our high ICU transfer rate, and prompt management of septic shock [37] may have contributed to limit mortality [2, 33, 38]. In rare cases, NEC was associated with severe infections, such as candidemia, with high mortality [36].

Conservative management has become the standard of care in most institutions [35]. Our data confirm the efficacy of a conservative approach, including broad-spectrum empiric antimicrobial therapy promptly started at the onset of fever [10] and general supportive care. Surgical management was reserved for situations of clinical deterioration despite optimal medical treatment, as proposed by Shamberger et al [14]. Indeed, a recent meta-analysis still favors surgery for patients with severe NEC requiring intensive care management [13].

This study has limitations, including its retrospective design and the fact that CT examination was decided by the treating physician. However, all patients were prospectively included, and data systematically reported in real-time. In auto-HCT patients, physicians may have been less prone to perform a CT scan, as neutropenia is of short duration and NEC is usually diagnosed just before neutrophil recovery. This may have contributed to underestimate the incidence of NEC in patients with auto-HCT with BEAM conditioning (14%), compared to the incidence observed in another study (31%), in which abdominal ultrasonography was performed systematically [34].

In conclusion, NEC is a common complication in neutropenic patients receiving standard AML induction chemotherapy or BEAM conditioning for auto-HCT. It requires close monitoring and abdominal CT examination in these groups, particularly in patients with AML with elevated circulating blast counts at diagnosis, high-dose corticosteroids, and/or previous NEC. Our results emphasize the effectiveness of medical management with a low 30-day mortality and the absence of a direct impact on long-term survival.

## Supplementary Material

ciaf134_Supplementary_Data

## Supplementary Data

Supplementary materials are available at *Clinical Infectious Diseases* online. Consisting of data provided by the authors to benefit the reader, the posted materials are not copyedited and are the sole responsibility of the authors, so questions or comments should be addressed to the corresponding author.

## Notes

***Author Contributions.*** P.-Y. B., C. S., and A.-S. B. designed the study. C. S. collected specific NEC data. S. A. and M. G. collected specific hematologic data. A.-S. B. designed the full clinical database under the supervision of P.-Y. B., with the contribution of O. M. for the period 2007–2014. M. F. drew up the treatment plans and validated the pharmacotherapy analyses. S. S. and K. M. reviewed all abdominal CT examinations. P.-Y. B. and A.-S. B. performed statistical analyses. P.-Y. B., A.-S. B., C. S., M. G., M. F., and O. S. contributed to the interpretation of the data. A.-S. B., P.-Y. B., and C. S. drafted the original manuscript. All authors revised and approved the final version of the manuscript.

***Acknowledgments.*** The authors thank the patients who participated in the study. They thank Galia Santos for her outstanding contribution to data collection. She tragically passed away at the beginning of this study. They acknowledge the contribution of Corine Guyat-Jacques for their important contribution in collecting clinical data as well as Fady Fares for data maintenance. They thank Eleftheria Kampouri for important advice for survival analyses.

***Data availability.*** Study data can be made available to investigators under specific data transfer agreement.

***Financial support.*** This work was supported by the Promex Stiftung für die Forschung (via Carigest SA) (to A.-S. B.); the Swiss National Science Foundation (grant numbers 31CA30_196036, 33IC30_179636, and 314730_192616) (to P.-Y. B.); the Leenaards Foundation to P.-Y. B.; and the Santos-Suarez Foundation (to P.-Y. B).
