# Supplementary material for: Epidemiology, Risk Factors, and Outcomes of Neutropenic Enterocolitis in Onco-Hematological Patients According to Chemotherapy Regimen
Source: Clin Infect Dis. 2025 Mar 20;82(2):e296–307. doi: 10.1093/cid/ciaf134 (PMC13017227; doi:10.1093/cid/ciaf134)
Supplement: ciaf134_Supplementary_Data [file ciaf134_supplementary_data.zip › SupplementaryTable1_EN_CID_final_06.01.2025.docx]

**Supplementary Table 1. Chemotherapeutic protocols.**

| **Name** | **AML** | | | | |
| --- | --- | --- | --- | --- | --- |
|  | **Induction AML** | | | | **Salvage AML** |
|  | **Standard induction - first induction (cycle I, “7+3”)** | | **Standard induction - second induction (cycle II)** | |  |
|  | ***Arm A*** | ***Arm B*** | ***Arm A*** | ***Arm B*** |  |
| **HOVON 042** | Ara-C 200 mg/m^2^/day D1-7 (METpyr)  Ida 12 mg/m^2^/day D5-7 (RUBI)  (+/- G-CSF D0-7) | Ara-C 200 mg/m^2^/day D1-7 (METpyr)  Ida 12 mg/m^2^/day D5-7 (RUBI)  (+/- G-CSF D0-7) | Ara-C 2000 mg/m^2^/day D1-6 (METpyr)  Amsa 120 mg/m^2^/day D3,5,7 (TopII)  (+/- G-CSF D0-7) | Ara-C 2000 mg/m^2^/day D1,2,4,6 (METpyr)  Amsa 120 mg/m^2^/day D3,5,7 (TopII)  (+/- G-CSF D0-7) |  |
| **HOVON 043** | Ara-C 200 mg/m^2^/day D1-7 (METpyr)  Dauno 45 mg/m^2^/day D1-3 (RUBI) | Ara-C 200 mg/m^2^/day D1-7 (METpyr)  Dauno 90 mg/m^2^/day D1-3 (RUBI) | Ara-C 2000 mg/m^2^/day D1-6 (METpyr) | Ara-C 2000 mg/m^2^/day D1-6 (METpyr) |  |
| **HOVON 081** | Ara-C 200 mg/m^2^/day D1-7 (METpyr)  Dauno 45 mg/m^2^/day D1-3 (RUBI) | Ara-C 200 mg/m^2^/day D1-7 (METpyr)  Dauno 45 mg/m^2^/day D1-3 (RUBI)  Bevacizumab^a^ D1,15 | Ara-C 2000 mg/m^2^/day D1-6 (METpyr) | Ara-C 2000 mg/m^2^/day D1-6 (METpyr)  Bevacizumab^a^ D1,15 |  |
| **HOVON 092** | Ara-C 200 mg/m^2^/day D1-7 (METpyr)  Ida 12 mg/m^2^/day D1-3 (RUBI) | Ara-C 200 mg/m^2^/day D1-7 (METpyr)  Ida 12 mg/m^2^/day D1-3 (RUBI)  Laromustine^a^ D2 | Ara-C 2000 mg/m^2^/day D1-6 (METpyr)  Amsa 120 mg/m^2^/day D4-6 (TopII) | Ara-C 2000 mg/m^2^/day D1-6 (METpyr)  Amsa 120 mg/m^2^/day D4-6 (TopII)  Laromustine^a^ D2 |  |
| **HOVON 102** | Ara-C 200 mg/m^2^/day D1-7 (METpyr)  Ida 12 mg/m^2^/day D1-3 (RUBI) | Ara-C 200 mg/m^2^/day D1-7 (METpyr)  Ida 12 mg/m^2^/day D1-3 (RUBI)  Clofarabine^a^ D1-5 (MET-PUR) | Ara-C 2000 mg/m^2^/day D1-6 (METpyr)  Amsa 120 mg/m^2^/day D4-6 (TopII) | Ara-C 2000 mg/m^2^/day D1-6 (METpyr)  Amsa 120 mg/m^2^/day D4-6 (TopII)  Clofarabine^a^ D1-5 (MET-PUR) |  |
| **HOVON 103** | ***Arm A*** | ***Arm B*** | ***Arm A*** | ***Arm B*** |  |
|  | Ara-C 200 mg/m^2^/day D1-7 (METpyr)  Dauno 60 mg/m^2^/day D1-3 (RUBI) | Ara-C 200 mg/m^2^/day D1-7 (METpyr)  Dauno 45 mg/m^2^/day D1-3 (RUBI)  Lenalidomide* D1-21 | Ara-C 2000 mg/m^2^/day D1-6 (METpyr) | Ara-C 2000 mg/m^2^/day D1-6 (METpyr)  Lenalidomide^a^ D1-21 |  |
|  | ***Arm C*** | ***Arm D*** | ***Arm C*** | ***Arm D*** |  |
|  | Ara-C 200 mg/m^2^/day D1-7 (METpyr)  Dauno 45 mg/m^2^/day D1-3 (RUBI)  Tosedostat^a^ D1-21 | Ara-C 200 mg/m^2^/day D1-7 (METpyr)  Dauno 60 mg/m^2^/day D1-3 (RUBI)  Selinexor^a^ twice weekly D1-24 | Ara-C 2000 mg/m^2^/day D1-6 (METpyr)  Tosedostat^a^ D1-56 | Ara-C 2000 mg/m^2^/day D1-6 (METpyr)  Selinexor^a^ twice weekly D1-24 |  |
| **HOVON 132** | Ara-C 200 mg/m^2^/day D1-7 (METpyr)  Ida 12 mg/m^2^/day D1-3 (RUBI) | Ara-C 200 mg/m^2^/day D1-7 (METpyr)  Ida 12 mg/m^2^/day D1-3 (RUBI)  Lenalidomide^a^ D1-21 | Ara-C 2000 mg/m^2^/day D1-6 (METpyr)  Dauno 60 mg/m^2^/day D1,3,5 (RUBI) | Ara-C 2000 mg/m^2^/day D1-6 (METpyr)  Dauno 60 mg/m^2^/day D1,3,5 (RUBI)  Lenalidomide^a^ D1-21 |  |
| **FLAG-ida** | Ida 8 mg/m^2^/day D1-3 (RUBI)  Fluda 30 mg/m^2^/day D1-5 (MET-PUR)  Ara-C^b^ 2000 mg/m^2^/day D1-5 (METpyr)  G-CSF D0-5 | | | | Ida 8 mg/m^2^/day D1-3 (RUBI)  Fluda 30 mg/m^2^/day D1-5 (MET-PUR)  Ara-C^b^ 2000 mg/m^2^/day D1-5 (METpyr)  G-CSF D0-5 |
| **CLAG-ida** | Ida 12 mg/m^2^/day D1-3 (RUBI)  Cladribine 5 mg/m^2^/day D1-5 (MET-PUR)  Ara-C^b^ 2000 mg/m^2^/day D1-5 (METpyr)  G-CSF D0-5 | | | | Ida 12 mg/m^2^/day D1-3 (RUBI)  Cladribine 5 mg/m^2^/day D1-5 (MET-PUR)  Ara-C^b^ 2000 mg/m^2^/day D1-5 (METpyr)  G-CSF D0-5 |

| **Name** | **ALL** | | |
| --- | --- | --- | --- |
|  | **Induction ALL** | | **Salvage reinduction** |
|  | **First induction** | |  |
|  | ***Standard-risk patient*** | ***High-risk patient (HyperC)*** |  |
| **GRALL 2005** | Prednisone 60 mg/m^2^/day D1-14  Vincristine 2 mg/day D1,8,15 and 22 (VIN)  Dauno 50 mg/m^2^/day D1-3 (RUBI)  Dauno 30 mg/m^2^/day D15-16 (RUBI)  L-asparaginase 6000 IU/m^2^/day D8,10,12,20,22,24,26 and 28 (Hydrol)  Cyclo 750 mg/m^2^/day D1 and 15 (ALK-Must)  G-CSF D18 to ANC>1G/L | Prednisone 60 mg/m^2^/day D1-14  Vincristine 2 mg/day D1,8,15 and 22 (VIN)  Dauno 50 mg/m^2^/day D1-3 (RUBI)  Dauno 30 mg/m^2^/day D15-16 (RUBI)  L- asparaginase 6000 IU/m^2^/day D8,10,12,20,22,24,26 and 28 (Hydrol)  Cyclo 750 mg/m^2^/day D1 (ALK-Must)  Cyclo 600 mg/m^2^/day D15-17 (ALK-Must)  G-CSF D18 to ANC>1G/L | Ida 12 mg/m^2^ /day D1-3 (RUBI)  Ara-C 4000 mg/m^2^/day D1-4 (METpyr)  G-CSF D9 to ANC>1G/L |
| **GRALL 2014** | Prednisone^c^ 60 mg/m^2^/day D1-14  Vincristine 2 mg/day D1,8,15 and 22 (VIN)  Dauno^c^ 50 mg/m^2^/day D1-3 (RUBI)  Dauno 30 mg/m^2^/day D15 and 16 (RUBI)  L- asparaginase^c^ 6000 IU/m^2^/day D8,10,12,20,22,24,26 and 28 (Hydrol)  Cyclo 750 mg/m^2^/day D1 and 15 (ALK-Must)  G-CSF D18 to ANC>1G/L |  |  |

| **Name** | **Hematopoietic Cell Transplantation (HCT)** |
| --- | --- |
| **BEAM**  (for lymphoma) | Carmustine 300 mg/m^2^/ day D-7 (ALK-Nitro)  Etoposide 200 mg/m^2^/ day D-6 to -3 (TopII)  Ara-C 400 mg/m^2^/ day D-6 to -3 (METpyr)  Melphalan 140 mg/m^2^/ day D-2 (ALK-Must) |
| **High dose melphalan**  (for multiple myeloma) | Melphalan 200 mg/m^2^/day D-2 (ALK-Must) (Cl_creat_ <30 ml/min 140 mg/m^2^/day D-2) |
| **BuCy**  (for AML) | Busulfan 3.2 mg/kg/day D-7 to -4 (ALK-Sulf)  Cyclo 60 mg/kg/day D-3 to -2 (ALK-Must) |
| **BuMel** | Busulfan 3.2 mg/kg/day D-7 to -3 (ALK-Sulf) (> 60-year-old 3.2 mg/kg/day D-7 to -4  Melphalan 140 mg/m^2^/day D-2 (ALK-Must) (> 60-year-old 120 mg/m^2^/day D-2) |
| **Carmustine thiotepa**  (for SNC lymphoma) | Carmustine 400 mg/m^2^/day D-6 (ALK-Nitro)  Thiotepa 5mg/kg/day D-5 to -4 (ALK-Thio) |

ALK-Must: alkylating agents, nitrogen mustards; ALK-Nitro : alkylating agents, nitrosurea; ALK-Sulf : alkylating agents, esters of alkane sulfonic acids; ALK-Thio: alkylating agents, ethyleneimine; ALL: acute lymphoid leukemia; AML: acute myeloid leukemia; Ara-C: Cytarabine; RUBI: antibiotic anthracycline; Cyclo : cyclophosphamide; D: day of administration; Dauno: daunorubicine; Fluda: fludarabine; HYDROL: hydrolyzes asparagine, deprives leukemia cells; Ida: Idarubicin; MET-PUR : antimetabolite, purine analogue and related inhibitor; METpyr: antimetabolite, pyrimidine analogues drug; TopII: inhibitors of type II topoisomerase and of religation of cleaved DNA strand; VIN: micro-tubule-damaging agents, vinca-alkaloids;

^a^ Assigned dose according to study protocols.

^b^ >60 year old adaptation: dose reduced to 1000mg mg/m^2^/d day 1-5

^c^ >45 year old adaptation: prednisone duration reduced to days 1-7, dauno reduced to 30 mg/m^2^, asparaginase omitted on day 26 and 28.
