# Supplementary material for: Epidemiology, Risk Factors, and Outcomes of Neutropenic Enterocolitis in Onco-Hematological Patients According to Chemotherapy Regimen
Source: Clin Infect Dis. 2025 Mar 20;82(2):e296–307. doi: 10.1093/cid/ciaf134 (PMC13017227; doi:10.1093/cid/ciaf134)
Supplement: ciaf134_Supplementary_Data [file ciaf134_supplementary_data.zip › SupplementaryTable2_EN_CID_final_ASB_29.11.2024.docx]

**Supplementary Table 2. Characteristics of population treated with ALL induction and other chemotherapies.**

|  |  | **Chemotherapy**  **for ALL** | |  | **Other**  **chemotherapies** | |
| --- | --- | --- | --- | --- | --- | --- |
| **Characteristics^a^** |  | **N** | **%** |  | **N** | **%** |
|  |  |  |  |  |  |  |
| **Number of chemotherapy courses** |  | 47 |  |  | 475 |  |
| **Number of patients** |  | 47 |  |  | 321 |  |
|  |  |  |  |  |  |  |
| **Patients demographics**^b^ |  |  |  |  |  |  |
| Median age (IQR) |  | 45 | (39) |  | 56 | (27) |
| Gender, Male |  | 25 | (53.2) |  | 189 | (58.9) |
| Ethnic, Caucasian |  | 44 | (93.6) |  | 300 | (93.5) |
|  |  |  |  |  |  |  |
| **Chronic health conditions**^b^ |  |  |  |  |  |  |
| Cardiac insufficiency |  | 2 | (4.3) |  | 37 | (11.5) |
| Pulmonary disease |  | 5 | (10.6) |  | 24 | (7.5) |
| Diabetes mellitus |  | 4 | (8.5) |  | 33 | (10.3) |
| Neurologic disease |  | 1 | (2.1) |  | 28 | (8.7) |
| Chronic renal insufficiency |  | 1 | (2.1) |  | 18 | (5.6) |
|  |  |  |  |  |  |  |
| **Underlying malignancy**^b^ |  |  |  |  |  |  |
| AML or MDS-IB2 |  | 1 | (2.1) |  | 152 | (47.4) |
| MM |  |  |  |  | 12 | (3.7) |
| Lymphoma |  |  |  |  | 45 | (14) |
| ALL |  | 40 | (85.1) |  | 66 | (20.6) |
| Other |  | 6 | (12.8) |  | 46 | (14.3) |
|  |  |  |  |  |  |  |
| **Chemotherapy regimens** |  |  |  |  |  |  |
| ALL - Induction |  | 47 | (100) |  |  |  |
| Other^c^ |  |  |  |  | 475 | (100) |
|  |  |  |  |  |  |  |
| Previous allogeneic HCT |  |  |  |  | 24 | (5.1) |
|  |  |  |  |  |  |  |
| Days in hospital (median, IQR) |  | 41 | (9) |  | 26 | (19) |
| Days of neutropenia (median, IQR) |  | 17 | (16) |  | 11 | (14) |
|  |  |  |  |  |  |  |
| Neutropenic enterocolitis |  | 4 | (8.5) |  | 8 | (1.7) |
|  |  |  |  |  |  |  |

ALL: acute lymphoblastic leukemia; AML: acute myeloid leukemia; HCT: hematopoietic cell transplantation; IQR: interquartile range; MDS-IB2: myelodysplastic syndrome with increased blasts 2; MM: multiple myeloma.

^a^ Continuous variables are described using medians and interquartile ranges and categorical variables using numbers and percentages (%).

^b^ Demographic characteristics, chronic health conditions and underlying malignancies are reported by patient (not by chemotherapy course).

^c^ Other chemotherapies included those administrated for multiple myeloma or non-Hodgkin’s lymphoma / Hodgkin’s lymphoma, or consolidation regimens for acute leukemia.
