# Supplementary material for: Epidemiology, Risk Factors, and Outcomes of Neutropenic Enterocolitis in Onco-Hematological Patients According to Chemotherapy Regimen
Source: Clin Infect Dis. 2025 Mar 20;82(2):e296–307. doi: 10.1093/cid/ciaf134 (PMC13017227; doi:10.1093/cid/ciaf134)
Supplement: ciaf134_Supplementary_Data [file ciaf134_supplementary_data.zip › SupplementaryTable3_EN_CID_final_ASB_PYB_06.01.2025.docx]

**Supplementary Table 3. Characteristics of 178 neutropenic enterocolitis episodes.**

|  | **All**  **NEC episodes**  **N=178** | |
| --- | --- | --- |
| **Variables^a^** | **N** | **(%)** |
|  |  |  |
| **Demographics** |  |  |
| Age, years (median, IQR) | 56 | (16) |
| Sex, male | 112 | (62.9) |
| **Chemotherapy regimens** |  |  |
| AML - Standard induction protocols^b^ | 75 | (42.1) |
| Purine-based chemotherapy^c^ | 17 | (9.6) |
| ALL - Induction | 4 | (2.2) |
| Auto-HCT - BEAM conditioning^d^ | 44 | (24.7) |
| Auto-HCT - other conditioning protocols^e^ | 30 | (16.9) |
| Other chemotherapies | 8 | (4.5) |
|  |  |  |
| Duration of neutropenia, days (median, IQR) | 14 | (15) |
| Delay between neutropenia and NEC, days (median, IQR) | 4 | (8) |
| Delay between admission and NEC, days (median, IQR) | 30 | (16) |
|  |  |  |
| **Concomitant infections^f^** |  |  |
| Bacteremia | 41 | (23.0) |
| Gram-negative bacilli | 23 | (12.9) |
| Gram-positive cocci | 20 | (11.2) |
| Anaerobes | 3 | (1.7) |
| Fungemia | 3 | (1.7) |
| *Candida albicans* | 1 | (0.6) |
| Hepatosplenic candidiasis | 2 | (1.1) |
| Invasive filamentous fungal infection | 11 | (6.2) |
| Pulmonary | 9 | (5.1) |
| Digestive | 2 | (1.1) |
|  |  |  |
| **Management** |  |  |
| ***Broad spectrum antibiotics****^g^* |  |  |
| Initial empirical antibiotic therapy (NEC as a first neutropenic fever) | 99 | (55.6) |
| Cefepime | 67 | (67.7) |
| Piperacillin-tazobactam | 23 | (23.2) |
| Carbapenem | 7 | (7.1) |
| Other (e.g. fluoroquinolone) | 2 | (2.0) |
|  |  |  |
| Antibiotic therapy at NEC diagnosis (new, modified or continued) | 178 | (100) |
| Cefepime/metronidazole | 74 | (41.6) |
| Piperacillin-tazobactam | 61 | (34.3) |
| Carbapenem | 39 | (21.9) |
| Other (e.g. fluoroquinolone) | 4 | (2.3) |
|  |  |  |
| ***Additional antibiotic therapy***^h^ |  |  |
| Vancomycin | 43 | (24.2) |
| Daptomycin | 5 | (2.8) |
| Aminoglycosides | 26 | (14.6) |
|  |  |  |
| ***Antifungal therapy***^i^ |  |  |
| Initial antifungal therapy at the time of NEC (if no previous antifungals) | 49 | (27.6) |
| Fluconazole | 33 | (30.6) |
| Voriconazole | 1 | (0.9) |
| Posaconazole/Isavuconazole | 2 | (1.9) |
| Echinocandins | 11 | (10.2) |
| Liposomal amphotericin B | 2 | (1.9) |
|  |  |  |
| Antifungal therapy at the NEC diagnosis (new, modified or continued) | 119 | (66.9) |
| Fluconazole | 60 | (33.7) |
| Voriconazole | 13 | (7.3) |
| Posaconazole/Isavuconazole | 19 | (10.7) |
| Echinocandins | 23 | (12.9) |
| Liposomal amphotericin B | 4 | (2.3) |
|  |  |  |
| G-CSF (within 10 days of NEC diagnosis) | 121 | (68.0) |
| Parenteral nutrition (within 10 days of NEC diagnosis) | 105 | (59.0) |
| Surgery | 2 | (1.1) |

ALL: acute lymphoblastic leukemia; AML: acute myeloid leukemia; CT: computed tomography; G-CSF: granulocyte-colony stimulating factor; auto-HCT: autologous hematopoietic cell transplant; IQR: interquartile range; NEC: neutropenic enterocolitis.

^a^ Continuous variables are described using medians and interquartile ranges, and categorical variables are described using numbers and proportions (%). Characteristics are reported by NEC episodes in a total of 168 patients.

^b^ All standard inductions regimens included standard-dose cytarabine with anthracyclines (mostly idarubicine or daunorubicine, “7+3”) for the first induction cycle and high-dose cytarabine +/- amsacrine or daunorubicin for the second induction cycle, according to the number of the HOVON/SAKK protocol [20].

^c^ Purine-based chemotherapy regimens included fludarabine, or seldomly cladribine, with high-dose cytarabine, and G-CSF +/- idarubicine for FLAG(-Ida), respectively CLAG(-Ida) protocols [21-25].

^d^ BEAM regimen included carmustine, etoposide, cytarabine and melphalan [15].

^e^ Other chemotherapeutic protocols included melphalan or other chemotherapies.

^f^ Within 2 days of the NEC diagnosis. Microbiological findings according to hospital stay are described in Table 6.

^g^ These drugs were either introduced as initial antibiotic therapy at the time of NEC (N=104), switched (N=61) or continued (N=13) from the initial treatment

^h^ These drugs were all prescribed at the time of NEC diagnosis, excepted for one case who already received daptomycin.

^I^ These drugs were either introduced as initial antifungal therapy at the time of NEC (N=49), switched (N=38) or continued (N=33) from the initial treatment.
