# Supplementary material for: Epidemiology, Risk Factors, and Outcomes of Neutropenic Enterocolitis in Onco-Hematological Patients According to Chemotherapy Regimen
Source: Clin Infect Dis. 2025 Mar 20;82(2):e296–307. doi: 10.1093/cid/ciaf134 (PMC13017227; doi:10.1093/cid/ciaf134)
Supplement: ciaf134_Supplementary_Data [file ciaf134_supplementary_data.zip › SupplementaryTable4_EN_CID_final_ASB_29.11.2024.docx]

**Supplementary Table 4. Risk factors for neutropenic enterocolitis in ALL.**

| **Characteristics**^a^ |  | **No NEC**  **(N=43)** | |  | **NEC**  **(N=4)** | |  | **Univariate** | | |  |
| --- | --- | --- | --- | --- | --- | --- | --- | --- | --- | --- | --- |
|  |  | **N** | **%** |  | **N** | **%** |  | **OR** | **(95%CI)** | **P** |  |
|  |  |  |  |  |  |  |  |  |  |  |  |
| **Age**, years (median, IQR) |  | 45 | (39) |  | 47 | (33) |  | 1.00 | (0.95-1.05) | 0.9 |  |
| **Gender**, male |  | 24 | (55.8) |  | 1 | (25.0) |  | 0.26 | (0.03-2.74) | 0.3 |  |
| **Ethnicity**, Caucasian |  | 40 | (93.0) |  | 4 | (100) |  |  |  |  |  |
|  |  |  |  |  |  |  |  |  |  |  |  |
| **Chronic health conditions** |  |  |  |  |  |  |  |  |  |  |  |
| Cardiac insufficiency |  | 2 | (4.7) |  |  |  |  |  |  |  |  |
| Pulmonary disease |  | 4 | (9.3) |  | 1 | (25.0) |  | 3.25 | (0.27-39.1) | 0.4 |  |
| Chronic renal failure |  | 1 | (2.3) |  |  |  |  |  |  |  |  |
| Neurological disease |  | 1 | (2.3) |  |  |  |  |  |  |  |  |
| Diabetes mellitus |  | 4 | (9.3) |  |  |  |  |  |  |  |  |
| Tobacco use |  | 9 | (20.9) |  | 1 | (25.0) |  | 1.26 | (0.12-13.60) | 0.8 |  |
|  |  |  |  |  |  |  |  |  |  |  |  |
| **Duration of agranulocytosis** |  |  |  |  |  |  |  |  |  |  |  |
| < 10 days |  | 3 | (7.0) |  |  |  |  |  |  |  |  |
| 11-25 days |  | 27 | (62.8) |  |  |  |  |  |  |  |  |
| > 25 days |  | 13 | (30.2) |  | 4 | (100) |  |  |  |  |  |
|  |  |  |  |  |  |  |  |  |  |  |  |
| **Other agents or conditions** |  |  |  |  |  |  |  |  |  |  |  |
| Corticosteroids > 100mg^b^ |  | 43 | (100) |  | 4 | (100) |  |  |  |  |  |
| G-CSF |  | 43 | (100) |  | 4 | (100) |  |  |  |  |  |
| Previous NEC |  | 43 | (100) |  | 4 | (100) |  |  |  |  |  |
|  |  |  |  |  |  |  |  |  |  |  |  |

ALL: acute lymphoblastic leukemia; CI: confidence interval; G-CSF: granulocyte-colony stimulating factor; IQR: interquartile range; NEC: Neutropenic enterocolitis; OR: Odd Ratio; Ref: Reference.

^a^ Continuous variables are described using medians and interquartile ranges, and categorical variables are described using numbers and proportions (%). Characteristics are reported by chemotherapy episode.

^b^ Total dose of corticosteroid during chemotherapy episode were calculated in prednisone equivalents : hydrocortisone (x 0.3), prednisolone (x 1), methylprednisolone (x 1.25), dexamethasone or betamethasone (x 6.7) [30].
