# Supplementary material for: Epidemiology, Risk Factors, and Outcomes of Neutropenic Enterocolitis in Onco-Hematological Patients According to Chemotherapy Regimen
Source: Clin Infect Dis. 2025 Mar 20;82(2):e296–307. doi: 10.1093/cid/ciaf134 (PMC13017227; doi:10.1093/cid/ciaf134)
Supplement: ciaf134_Supplementary_Data [file ciaf134_supplementary_data.zip › SupplementaryTable5_EN_CID_final_ASB_29.11.2024.docx]

**Supplementary Table 5. Risk factors of neutropenic enterocolitis for other chemotherapies.**

| **Characteristics**^a^ | **No NEC**  **(N=467)** | |  | **NEC**  **(N=8)** | |  | **Univariate** | | |  | **Multivariate^c^**  **(N=319)** | | |
| --- | --- | --- | --- | --- | --- | --- | --- | --- | --- | --- | --- | --- | --- |
|  | **N** | **%** |  | **N** | **%** |  | **OR** | **(95%CI)** | **P** |  | **OR** | **(95%CI)** | **P** |
|  |  |  |  |  |  |  |  |  |  |  |  |  |  |
| **Age**, years (median, IQR) | 51.7 | (28.8) |  | 63 | (24.2) |  | 1.01 | (0.97-1.06) | 0.5 |  |  |  |  |
| **Gender**, male | 271 | (58.0) |  | 6 | (75) |  | 2.17 | (0.43-10-86) | 0.3 |  |  |  |  |
| **Ethnicity**, Caucasian | 433 | (92.7) |  | 8 | (100) |  |  |  |  |  |  |  |  |
|  |  |  |  |  |  |  |  |  |  |  |  |  |  |
| **Chronic health conditions** |  |  |  |  |  |  |  |  |  |  |  |  |  |
| Cardiac insufficiency | 49 | (10.5) |  | 3 | (37.5) |  | 5.12 | (1.19-22.08) | **0.029** |  |  |  |  |
| Pulmonary disease | 34 | (7.3) |  | 1 | (12.5) |  | 1.82 | (0.22-15.22) | 0.6 |  |  |  |  |
| Chronic renal failure | 19 | (4.1) |  | 1 | (12.5) |  | 3.37 | (0.39-28.77) | 0.3 |  |  |  |  |
| Neurological disease | 38 | (8.1) |  | 0 |  |  |  |  |  |  |  |  |  |
| Diabetes mellitus | 43 | (9.2) |  | 1 | (12.5) |  | 1.41 | (0.17-11.72) | 0.8 |  |  |  |  |
| Tobacco use | 98 | (21.0) |  | 2 | (25.0) |  | 1.26 | (0.25-6.32) | 0.8 |  |  |  |  |
|  |  |  |  |  |  |  |  |  |  |  |  |  |  |
| **Chemotherapy regimen** |  |  |  |  |  |  |  |  |  |  |  |  |  |
| AML consolidation with azacitidine | 122 | (26.1) |  | 3 | (37.5) |  | Ref. |  |  |  |  |  |  |
| Philadelphia positive B-ALL | 27 | (5.8) |  | 1 | (12.5) |  | 1.51 | (0.15-15.04) | 0.7 |  |  |  |  |
| ALL (other than induction) | 113 | (24.2) |  | 0 |  |  |  |  |  |  |  |  |  |
| Lymphoma or MM | 162 | (34.7) |  | 4 | (50) |  | 1.00 | (0.22-4.57) | 1.00 |  |  |  |  |
| Other | 43 | (9.2) |  | 0 |  |  |  |  |  |  |  |  |  |
|  |  |  |  |  |  |  |  |  |  |  |  |  |  |
| **Duration of agranulocytosis** |  |  |  |  |  |  |  |  |  |  |  |  |  |
| <10 days | 224 | (48.0) |  | 2 | (25.0) |  | Ref. |  |  |  |  |  |  |
| 11-25 days | 171 | (36.6) |  | 3 | (37.5) |  | 1.96 | (0.32-11.89) | 0.5 |  |  |  |  |
| >25 days | 72 | (15.4) |  | 3 | (37.5) |  | 4.67 | (0.76-28.48) | 0.1 |  |  |  |  |
|  |  |  |  |  |  |  |  |  |  |  |  |  |  |
| **Other agents or conditions** |  |  |  |  |  |  |  |  |  |  |  |  |  |
| Corticosteroids > 100mg^b^ | 334 | (71.5) |  | 4 | (50.0) |  | 0.40 | (0.10-1.62) | 0.2 |  |  |  |  |
| G-CSF | 381 | (81.6) |  | 6 | (75.0) |  | 0.68 | (0.13-3.41) | 0.6 |  |  |  |  |
| Previous NEC | 27 | (6.0) |  | 2 | (25.0) |  | 5.43 | (1.05-28.20) | **0.044** |  | 5.76 | (1.08-30.73) | **<0.001** |
|  |  |  |  |  |  |  |  |  |  |  |  |  |  |

ALL: acute lymphoblastic leukemia; AML: acute myeloid leukemia; CI: confidence interval; G-CSF: granulocyte-colony stimulating factor; IQR: interquartile range; MM: multiple myeloma; NEC: Neutropenic enterocolitis; OR: Odd Ratio; Ref: Reference.

^a^ Continuous variables are described using medians and interquartile ranges, and categorical variables are described using numbers and proportions (%). Characteristics are reported by chemotherapy episode.

^b^ Total dose of corticosteroid during chemotherapy episode were calculated in prednisone equivalents : hydrocortisone (x 0.3), prednisolone (x 1), methylprednisolone (x 1.25), dexamethasone or betamethasone (x 6.7) [30].

^c^ Variables with a P value <0.2 were entered into multivariable models and subsequently selected by using the stepwise program implemented in Stata^®^, with backward removal of variable with a P value <0.1.
