# Supplementary material for: Epidemiology, Risk Factors, and Outcomes of Neutropenic Enterocolitis in Onco-Hematological Patients According to Chemotherapy Regimen
Source: Clin Infect Dis. 2025 Mar 20;82(2):e296–307. doi: 10.1093/cid/ciaf134 (PMC13017227; doi:10.1093/cid/ciaf134)
Supplement: ciaf134_Supplementary_Data [file ciaf134_supplementary_data.zip › SupplementaryTable6_EN_CID_final_ASB_PYB_29.11.2024.docx]

**Supplementary Table 6. Comparative outcomes of patients with and without neutropenic enterocolitis according to chemotherapy regimens.**

|  | **Standard AML induction** | | | | | | |  |  | **Purine-based chemotherapy for AML** | | | | | | |  | **HCT - BEAM** | | | | | | |  |
| --- | --- | --- | --- | --- | --- | --- | --- | --- | --- | --- | --- | --- | --- | --- | --- | --- | --- | --- | --- | --- | --- | --- | --- | --- | --- |
|  |  | **No NEC** | |  | **NEC** | |  |  |  |  | **No NEC** | |  | **NEC** | |  |  |  | **No NEC** | |  | **NEC** | |  |  |
| **Characteristics^a^** | **N** | **N** | **%** |  | **N** | **%** | **P** |  |  | **N** | **N** | **%** |  | **N** | **%** | **P** |  | **N** | **N** | **%** |  | **N** | **%** | **P** |  |
|  |  |  |  |  |  |  |  |  |  |  |  |  |  |  |  |  |  |  |  |  |  |  |  |  |  |
| **Chemotherapy episodes** | 411 | 336 |  |  | 75 |  |  |  |  | 254 | 237 |  |  | 17 |  |  |  | 289 | 245 |  |  | 44 |  |  |  |
|  |  |  |  |  |  |  |  |  |  |  |  |  |  |  |  |  |  |  |  |  |  |  |  |  |  |
| **Days in hospital,** median (IQR) |  | 33 | (19) |  | 34 | (21) | 0.3 |  |  |  | 30 | (13.5) |  | 38 | (28) | 0.1 |  |  | 22 | (5) |  | 27 | (6) | **0.006** |  |
|  |  |  |  |  |  |  |  |  |  |  |  |  |  |  |  |  |  |  |  |  |  |  |  |  |  |
| **Associated infections^b^** |  |  |  |  |  |  |  |  |  |  |  |  |  |  |  |  |  |  |  |  |  |  |  |  |  |
| Bacteremia | 208 | 171 | (50.9) |  | 37 | (49.3) | 0.8 |  |  | 137 | 124 | (52.3) |  | 13 | (76.5) | 0.06 |  | 64 | 53 | (21.6) |  | 11 | (25) | 0.6 |  |
| Gram-negative bacilli | 87 | 71 | (21.1) |  | 16 | (21.3) | 0.9 |  |  | 61 | 57 | (24.1) |  | 4 | (23.5) | 0.9 |  | 34 | 30 | (12.2) |  | 4 | (9.1) | 0.6 |  |
| Gram-positive cocci | 112 | 87 | (25.9) |  | 25 | (33.3) | 0.2 |  |  | 70 | 60 | (25.3) |  | 10 | (58.8) | **0.005** |  | 24 | 19 | (7.8) |  | 5 | (11.4) | 0.4 |  |
| Anaerobes | 13 | 12 | (3.6) |  | 1 | (1.3) | 0.3 |  |  | 9 | 9 | (3.8) |  |  |  |  |  | 3 | 2 | (0.8) |  | 1 | (2.3) | 0.4 |  |
| Polymicrobial | 94 | 78 | (23.2) |  | 16 | (21.3) | 0.7 |  |  | 67 | 63 | (26.6) |  | 4 | (23.5) | 0.8 |  | 37 | 32 | (13.1) |  | 5 | (11.4) | 0.8 |  |
| Fungemia | 7 | 3 | (0.9) |  | 4 | (5.3) | **0.02** |  |  | 3 | 1 | (0.4) |  | 2 | (11.8) | **0.006** |  | 6 | 3 | (1.2) |  | 3 | (6.8) | **0.03** |  |
| Hepatosplenic candidiasis^c^ | 10 | 8 | (2.4) |  | 2 | (2.7) | 0.9 |  |  | 1 | 1 | (0.4) |  |  |  |  |  |  |  |  |  |  |  |  |  |
| Invasive filamentous fungal infection^d^ |  |  |  |  |  |  |  |  |  |  |  |  |  |  |  |  |  |  |  |  |  |  |  |  |  |
| Pulmonary localization | 30 | 21 | (6.2) |  | 9 | (12) | 0.1 |  |  | 21 | 19 | (8) |  | 2 | (11.8) | 0.6 |  |  |  |  |  |  |  |  |  |
| Digestive localization | 2 | 1 | (0.1) |  | 1 | (0.6) | 0.1 |  |  |  |  |  |  |  |  |  |  |  |  |  |  |  |  |  |  |
|  |  |  |  |  |  |  |  |  |  |  |  |  |  |  |  |  |  |  |  |  |  |  |  |  |  |
| **Worse severity scores** |  |  |  |  |  |  |  |  |  |  |  |  |  |  |  |  |  |  |  |  |  |  |  |  |  |
| Quick SOFA^f^ ≥ 2 | 53 | 38 | (11.3) |  | 15 | (20) | **0.045** |  |  | 30 | 27 | (11.4) |  | 3 | (17.6) | 0.5 |  | 26 | 14 | (5.7) |  | 12 | (27.3) | **<0.001** |  |
| SOFA^g^ |  |  |  |  |  |  |  |  |  |  |  |  |  |  |  |  |  |  |  |  |  |  |  |  |  |
| <5 | 373 | 303 | (90.2) |  | 70 | (93.3) | Ref. |  |  | 222 | 208 | (87.8) |  | 14 | (82.4) | Ref. |  | 266 | 232 | (94.7) |  | 34 | (77.3) | Ref. |  |
| 5-10 | 34 | 31 | (9.2) |  | 3 | (4) | 0.2 |  |  | 26 | 25 | (10.5) |  | 1 | (5.9) | 0.6 |  | 18 | 11 | (4.5) |  | 7 | (15.9) | **0.005** |  |
| >10 | 4 | 2 | (0.6) |  | 2 | (2.7) | 0.2 |  |  | 6 | 4 | (1.7) |  | 2 | (11.8) | **0.03** |  | 5 | 2 | (0.8) |  | 3 | (6.8) | **0.01** |  |
|  |  |  |  |  |  |  |  |  |  |  |  |  |  |  |  |  |  |  |  |  |  |  |  |  |  |
| **Transfer to ICU^h^** | 35 | 26 | (7.9) |  | 9 | (12) | 0.3 |  |  | 20 | 16 | (7.1) |  | 4 | (23.5) | **0.03** |  | 18 | 5 | (2.1) |  | 13 | (29.1) | **<0.001** |  |
|  |  |  |  |  |  |  |  |  |  |  |  |  |  |  |  |  |  |  |  |  |  |  |  |  |  |
| **Abdominal surgery** | 6 | 5 | (1.5) |  | 2 | (2.7) | 0.5 |  |  | 1 |  |  |  | 1 | (5.9) | - |  | 1 |  |  |  | 1 | (2.3) |  |  |
|  |  |  |  |  |  |  |  |  |  |  |  |  |  |  |  |  |  |  |  |  |  |  |  |  |  |
| **In hospital all-causes mortality^i^** | 11 | 8 | (2.4) |  | 3 | (4) | 0.4 |  |  | 9 | 6 | (2.5) |  | 3 | (17.6) | **0.005** |  | 4 | 3 | (1.2) |  | 1 | (2.3) | 0.6 |  |
|  |  |  |  |  |  |  |  |  |  |  |  |  |  |  |  |  |  |  |  |  |  |  |  |  |  |

**Supplementary Table 6. Comparative outcomes of patients with and without neutropenic enterocolitis according to chemotherapy regimens, continued.**

|  | **HCT – other than BEAM** | | | | | | |  |  | **Induction ALL** | | | | | | |  | **Other chemotherapies** | | | | | | |  |
| --- | --- | --- | --- | --- | --- | --- | --- | --- | --- | --- | --- | --- | --- | --- | --- | --- | --- | --- | --- | --- | --- | --- | --- | --- | --- |
|  |  | **No NEC** | |  | **NEC** | |  |  |  |  | **No NEC** | |  | **NEC** | |  |  |  | **No NEC** | |  | **NEC** | |  |  |
| **Characteristics^a^** | **N** | **N** | **%** |  | **N** | **%** | **P** |  |  | **N** | **N** | **%** |  | **N** | **%** | **P** |  | **N** | **N** | **%** |  | **N** | **%** | **P** |  |
|  |  |  |  |  |  |  |  |  |  |  |  |  |  |  |  |  |  |  |  |  |  |  |  |  |  |
| **Chemotherapy episodes** | 487 | 457 |  |  | 30 |  |  |  |  | 47 | 43 |  |  | 4 |  |  |  | 475 | 467 |  |  | 8 |  |  |  |
|  |  |  |  |  |  |  |  |  |  |  |  |  |  |  |  |  |  |  |  |  |  |  |  |  |  |
| **Days in hospital,** median (IQR) |  | 19 | (5.5) |  | 20 | (20) | **0.02** |  |  |  | 42 | (6) |  | 55 | (54) | **0.001** |  |  | 27 | (19.5) |  | 29 | (15.8) | **0.02** |  |
|  |  |  |  |  |  |  |  |  |  |  |  |  |  |  |  |  |  |  |  |  |  |  |  |  |  |
| **Associated infections^b^** |  |  |  |  |  |  |  |  |  |  |  |  |  |  |  |  |  |  |  |  |  |  |  |  |  |
| Bacteremia | 110 | 104 | (22.8) |  | 6 | (20) | 0.7 |  |  | 22 | 18 | (41.9) |  | 4 | (100) | - |  | 116 | 112 | (24) |  | 4 | (50) | 0.1 |  |
| Gram-negative bacilli | 59 | 55 | (12) |  | 4 | (13.3) | 0.8 |  |  | 9 | 8 | (18.6) |  | 1 | (25) | 0.8 |  | 53 | 49 | (10.5) |  | 4 | (50) | **0.003** |  |
| Gram-positive cocci | 39 | 37 | (8.1) |  | 2 | (6.7) | 0.8 |  |  | 8 | 5 | (11.6) |  | 3 | (75) | **0.01** |  | 65 | 63 | (13.5) |  | 2 | (25) | 0.4 |  |
| Anaerobes | 2 | 2 | (0.4) |  |  |  |  |  |  |  |  |  |  |  |  |  |  | 12 | 12 | (2.6) |  |  |  |  |  |
| Polymicrobial | 60 | 56 | (12.3) |  | 4 | (13.3) | 0.9 |  |  | 9 | 8 | (18.6) |  | 1 | (25) | 0.8 |  | 54 | 50 | (10.7) |  | 4 | (50) | **0.003** |  |
| Fungemia | 1 | 1 | (0.2) |  |  |  |  |  |  |  |  |  |  |  |  |  |  | 3 | 2 | (0.4) |  | 1 | (12.5) | **0.006** |  |
| Hepatosplenic candidiasis^c^ | 0 |  |  |  |  |  |  |  |  |  |  |  |  |  |  |  |  | 4 | 3 | (0.6) |  | 1 | (12.5) | **0.01** |  |
| Invasive filamentous fungal infection^d^ |  |  |  |  |  |  |  |  |  |  |  |  |  |  |  |  |  |  |  |  |  |  |  |  |  |
| Pulmonary localization | 1 | 1 | (0.2) |  |  |  |  |  |  | 4 | 4 | (9.3) |  |  |  |  |  | 15 | 14 | (3) |  | 1 | (12.5) | 0.2 |  |
| Digestive localization |  |  |  |  |  |  |  |  |  |  |  |  |  |  |  |  |  |  |  |  |  |  |  |  |  |
|  |  |  |  |  |  |  |  |  |  |  |  |  |  |  |  |  |  |  |  |  |  |  |  |  |  |
| **Worse severity scores** |  |  |  |  |  |  |  |  |  |  |  |  |  |  |  |  |  |  |  |  |  |  |  |  |  |
| Quick SOFA^f^ ≥ 2 | 29 | 24 | (5.3) |  | 5 | (16.7) | **0.02** |  |  | 5 | 5 | (11.6) |  |  |  |  |  | 40 | 37 | (7.9) |  | 3 | (37.5) | **0.01** |  |
| SOFA^g^ |  |  |  |  |  |  |  |  |  |  |  |  |  |  |  |  |  |  |  |  |  |  |  |  |  |
| <5 | 468 | 442 | (96.7) |  | 26 | (86.7) | Ref. |  |  | 45 | 41 | (95.3) |  | 4 | (100) | Ref. |  | 443 | 437 | (93.6) |  | 6 | (75) | Ref. |  |
| 5-10 | 18 | 14 | (3.1) |  | 4 | (13.3) | **0.009** |  |  |  |  |  |  |  |  |  |  | 29 | 28 | (6) |  | 1 | (12.5) | 0.4 |  |
| >10 | 1 | 1 | (0.2) |  |  |  |  |  |  | 2 | 2 | (4.7) |  |  |  |  |  | 3 | 2 | (0.4) |  | 1 | (12.5) | **0.005** |  |
|  |  |  |  |  |  |  |  |  |  |  |  |  |  |  |  |  |  |  |  |  |  |  |  |  |  |
| **Transfer to ICU^h^** | 10 | 9 | (2.1) |  | 1 | (3.3) | 0.7 |  |  | 4 | 3 | (8.1) |  | 1 | (25) | 0.3 |  | 13 | 11 | (3) |  | 2 | (25) | **0.007** |  |
|  |  |  |  |  |  |  |  |  |  |  |  |  |  |  |  |  |  |  |  |  |  |  |  |  |  |
| **Abdominal surgery** |  |  |  |  |  |  |  |  |  |  |  |  |  |  |  |  |  | 3 | 3 | (0.6) |  |  |  |  |  |
|  |  |  |  |  |  |  |  |  |  |  |  |  |  |  |  |  |  |  |  |  |  |  |  |  |  |
| **In hospital all-causes mortality^i^** |  |  |  |  |  |  |  |  |  | 2 | 2 | (4.7) |  |  |  |  |  | 29 | 28 | (6) |  | 1 | (12.5) | 0.5 |  |
|  |  |  |  |  |  |  |  |  |  |  |  |  |  |  |  |  |  |  |  |  |  |  |  |  |  |

ICU: intensive care unit; IQR: interquartile range; NEC: Neutropenic enterocolitis; ref: Reference; SOFA: sequential organ failure assessment.

^a^ Continuous variables are described using medians and interquartile ranges, and categorical variables are described using numbers and proportions (%). Characteristics are reported by NEC episodes.

^b^ Infections identified during the entire hospital stay.

^c^ Concomitant fungemia was identified for 2 of the 10 episodes in the standard induction AML and 2 of the 4 episodes in other chemotherapies group. No case was observed for the other groups.

^d^ Among invasive filamentous fungal infections, the number of invasive aspergilloses was 27/34 in induction AML, 16/25 in purine-based chemotherapy for AML, 1/1 in HCT with no BEAM conditioning, 4/4 in ALL induction and 15/18 in other chemotherapies.

^e^ Sepsis score according Sepsis-3 definitions [28]; patients who did not develop fever and/or symptoms suggestive of sepsis were allocated the minimal score. Missing data: 140 in standard induction AML, 161 in purine-based chemotherapy for AML, 118 in HCT with BEAM group, 258 in HCT without BEAM group, 24 in induction ALL, and 239 in other chemotherapies.

^f^ The QuickSOFA score [28] ranged from 0 to 3 according to presence or absence of these criteria: systolic blood pressure ≤100mmHg, tachypnea ≥22 breath per min and Glasgow coma score ≤14 (one point each); patients who did not develop fever and/or symptoms suggestive of sepsis were allocated the minimal score.

^g^ Patient who did not develop fever and/or symptoms suggestive of sepsis are in the lower score group (<5).

^h^ All-causes of ICU admission during the hospital course. Missing data: 8 in standard induction AML, 12 in purine-based chemotherapy for AML, 2 in HCT - BEAM group, 29 in HCT – no BEAM group, 6 in induction ALL and 102 in other chemotherapies.

^i^ All-causes of mortality; see Figure 2 for Kaplan-Maier survival estimates.
